# CRISPR-Cas Diversity in Clinical *Salmonella enterica* Serovar Typhi Isolates from South Asian Countries

**DOI:** 10.3390/genes11111365

**Published:** 2020-11-18

**Authors:** Arif Mohammad Tanmoy, Chinmoy Saha, Mohammad Saiful Islam Sajib, Senjuti Saha, Florence Komurian-Pradel, Alex van Belkum, Rogier Louwen, Samir Kumar Saha, Hubert P. Endtz

**Affiliations:** 1 Department of Medical Microbiology and Infectious Diseases, Erasmus University Medical Center Rotterdam, 3015 CN Rotterdam, The Netherlands; arif.tanmoy@chrfbd.org (A.M.T.); c.saha@erasmusmc.nl (C.S.); hubert.endtz@fondation-merieux.org (H.P.E.); 2 Child Health Research Foundation, 23/2 SEL Huq Skypark, Block-B, Khilji Rd, Dhaka 1207, Bangladesh; saiful.i.saijb@chrfbd.org (M.S.I.S.); senjutisaha@chrfbd.org (S.S.); samir@chrfbd.org (S.K.S.); 3 Laboratoire des Pathogènes Emergents, Fondation Mérieux, Centre International de Recherche en Infectiologie (CIRI), INSERM U1111, 69365 Lyon, France; florence.pradel@fondation-merieux.org; 4 Data Analytics Unit, bioMérieux, 3, Route de Port Michaud, 38390 La Balme Les Grottes, France; alex.vanbelkum@biomerieux.com; 5 Bangladesh Institute of Child Health, Dhaka Shishu Hospital, Dhaka 1207, Bangladesh

**Keywords:** *Salmonella* Typhi, CRISPR diversity, *cas* genes, antibiotic resistance, Typhi PAM, spacer targets

## Abstract

Typhoid fever, caused by *Salmonella enterica* serovar Typhi (*S.* Typhi), is a global health concern and its treatment is problematic due to the rise in antimicrobial resistance (AMR). Rapid detection of patients infected with AMR positive *S.* Typhi is, therefore, crucial to prevent further spreading. *C*lustered *R*egularly *I*nterspaced *S*hort *P*alindromic *R*epeats and CRISPR-associated genes (CRISPR-Cas), is an adaptive immune system that initially was used for typing purposes. Later, it was discovered to play a role in defense against phages and plasmids, including ones that carry AMR genes, and, at present, it is being explored for its usage in diagnostics. Despite the availability of whole-genome sequences (WGS), very few studied the CRISPR-Cas system of *S.* Typhi, let alone in typing purposes or relation to AMR. In the present study, we analyzed the CRISPR-Cas system of *S.* Typhi using WGS data of 1059 isolates obtained from Bangladesh, India, Nepal, and Pakistan in combination with demographic data and AMR status. Our results reveal that the *S.* Typhi CRISPR loci can be classified into two groups: A (evidence level >2) and B (evidence level ≤2), in which we identified a total of 47 unique spacers and 15 unique direct repeats. Further analysis of the identified spacers and repeats demonstrated specific patterns that harbored significant associations with genotype, demographic characteristics, and AMR status, thus raising the possibility of their usage as biomarkers. Potential spacer targets were identified and, interestingly, the phage-targeting spacers belonged to the group-A and plasmid-targeting spacers to the group-B CRISPR loci. Further analyses of the spacer targets led to the identification of an *S.* Typhi protospacer adjacent motif (PAM) sequence, TTTCA/T. New *cas*-genes known as *DinG*, *DEDDh*, and *WYL* were also discovered in the *S.* Typhi genome. However, a specific variant of the *WYL* gene was only identified in the extensively drug-resistant (XDR) lineage from Pakistan and ciprofloxacin-resistant lineage from Bangladesh. From this work, we conclude that there are strong correlations between variations identified in the *S.* Typhi CRISPR-Cas system and endemic AMR positive *S.* Typhi isolates.

## 1. Introduction

Typhoid fever is a systemic enteric infection, caused by *Salmonella enterica* serovar Typhi (*S.* Typhi), a human-restricted bacterial pathogen [1,2]. It is estimated to lead to 117 thousand deaths and 11 million episodes of illnesses every year and thus remains a major global public health concern [3]. The fecal–oral transmission route of *S.* Typhi makes typhoid fever highly endemic in areas with poor water and sanitation systems, especially the South Asian countries such as Bangladesh, India, Nepal, and Pakistan [3,4]. Moreover, treating typhoid fever has become harder, because of the increasing antimicrobial resistance (AMR) [5]. Recently, a highly clonal and extensively drug-resistant (XDR) lineage of *S.* Typhi that is resistant to all, but one oral antibiotic, azithromycin, caused a large-scale typhoid outbreak in Pakistan [6]. A highly ciprofloxacin-resistant lineage (named ‘Bdq’; as a part of genotype 4.3.1.3, it will be referred to as 4.3.1.3q1 in the rest of the article) has appeared in Bangladesh and carries a *qnr* gene-containing plasmid, pK91 [5,7]. Isolates with high azithromycin resistance have been reported in Bangladesh as well [8,9]. With the availability of whole-genome sequence (WGS) data, these AMR characteristics can be easily detected and a large amount of WGS data is publicly available for *S.* Typhi. WGS data can also shed light on the presence of defense mechanisms that can recognize and destroy foreign genetic materials [10].

One such system is the *C*lustered *R*egularly *I*nterspaced *S*hort *P*alindromic *R*epeat and CRISPR-associated genes (CRISPR-Cas) for which little information is available in *S.* Typhi [11,12,13,14]. A CRISPR locus usually contains two to several hundreds of direct repeat (DR) sequences of 23–50 bp in length, separated by unique spacer sequences of similar length [15]. Spacers share complementarity with sequences identified in foreign DNA elements (protospacers) and are acquired from phages, plasmids, and other transferrable elements that previously infected bacteria [16,17,18]. To differentiate foreign DNA elements from self-DNA, the Cas proteins follow often at least three-nucleotide long protospacer-adjacent motif (PAM) present on the target sequence [19,20].

The genus *Salmonella* is known to carry a class-1 type I-E system, closely related to the CRISPR-Cas system in *Escherichia coli* (*E*. *coli*) [21,22]. The systems have been reported to carry either one or two CRISPR loci and a *cas*-gene cluster of *cas3*, *cse1*-*cse2*-*cas7*-*cas5*-*cas6e*-*cas1*-*cas2* genes [2,14]. CRISPR-Cas systems in other bacterial species have been explored extensively for typing purposes [23]. For AMR, it became evident that the size of the CRISPR loci correlates with the presence or absence of AMR-related genes [24,25,26,27]. In *S*. Typhi, only a few studies explored the usage of the CRISPR-Cas system for typing purposes, which is still an unexplored territory [11,12]. Moreover, the earlier studies analyzed only a smaller number of whole-genome sequences (WGS) to explore the diversity of the system. For example, Fabre et al. used 18 *S.* Typhi WGS data to report two different CRISPR loci in the genome (CRISPR1 and CRISPR2) and used PCR assays to amplify those loci to explore the diversity of DR and spacers [11]. Therefore, an opportunity exists to follow-up this work with a larger set of WGS data to explore the *S.* Typhi CRISPR-Cas system further and report on its diversity as well.

In this work, we analyzed the *S.* Typhi CRISPR-Cas system using WGS data of 1059 isolates obtained from four major typhoid-endemic countries (Bangladesh, India, Nepal and Pakistan) with the country of isolation, demographic data, and AMR status. Our work identified potential CRISPR-Cas system-related markers that associate specifically with endemic and AMR-related S. Typhi isolates. We further identified unique spacer targets in bacteriophages and plasmids that led to the identification of a specific PAM sequence for *S.* Typhi. Next, we annotated common and new *cas* genes, of which one, the gene *WYL*, could be specifically linked to XDR isolates from Pakistan. Collectively, our study reveals with an impressive dataset that the CRISPR-Cas system in *S.* Typhi might become of use to monitor the dissemination of AMR endemic isolates so that their spreading can be contained.

## 2. Materials and Methods

### 2.1. Source and Assembly of the Genome Data

We used published WGS data of 536 isolates from Bangladesh, 198 from Nepal, 131 from India, and 20 from Pakistan [5,28,29]. These 885 isolates were considered as “Surveillance” cases. WGS data of 100 isolates from the ongoing XDR *S.* Typhi outbreak in Pakistan were included and considered as “Outbreak” cases [6]. Moreover, we included WGS of 74 travel-associated typhoid cases from the UK who traveled from the four above mentioned countries and categorized them as “Travel” cases [30]. Details of all 1059 cases are provided in Dataset S1. Raw *S.* Typhi genome data (fastq files) of all cases were downloaded from the European Nucleotide Archive (ENA), following the accessions given in source articles. We used SPAdes v3.12.0 (options: cov-cutoff = ’auto’) to assemble the fastq files and removed smaller contigs (<300 bp) [31]. N50 of the contig files were calculated and added in the Dataset S1.

To compare the *S*. Typhi isolates with other *Salmonella* serovars, we added sequences of 48 complete chromosomes of 19 different *Salmonella* serovars (excluding *S.* Typhi) from NCBI-genome (https://www.ncbi.nlm.nih.gov/genome/genomes/152, downloaded on 12 October 2018). We also included six representative reference genomes of *E. coli* (https://www.ncbi.nlm.nih.gov/genome/genomes/167, downloaded on 12 October 2018). Accession numbers of all 54 complete chromosomes of different *Salmonella* species/serovars and *E. coli* isolates are listed in Dataset S2.

### 2.2. Detection of CRISPR Loci and Cas Genes

To detect the CRISPR loci, we ran all assembled contigs through the CRISPRCasFinder v4.2.19 locally (without the *cas*-gene option) [32]. Following the earlier reports of *S.* Typhi DR and spacer length (29 and 32 bp) [11,21], all DRs and spacers longer than that were checked manually for truncation and overlap. Identified confirmed loci with an evidence score of 3 or 4 harbored increased numbers of spacers and were considered as “group-A CRISPR loci”, which is the same as CRISPR1, an earlier nomenclature used by Fabre et al. [11]. However, unlike previous reports, more than one locus with an evidence score of 1 or 2 (low number of spacers) were found and all can be compared to CRISPR2 of the earlier nomenclature, thus we considered them as part of the second group of loci, “group-B CRISPR loci”.

Sequences of direct repeats (DR) and spacers were extracted from all *S.* Typhi, *Salmonella* spp. and *E. coli* isolates separately and screened for sequence identity within their groups (ignored if redundant otherwise termed as ‘unique’). All unique DR and spacers were given a unique three-part identifier (e.g., Td29a, Ts32ac, Ss32aak) following the strategy explained in Figure S1. Spacer arrangements of all group-A and B CRISPR loci were determined separately.

To detect the *cas* genes, we used Prokka v1.13.3 (options: gcode = 11) to annotate all 1113 genomes and blastp v2.7.1 + (options: evalue = 1 × 10^−9^ and qcov_hsp_perc = 80) to search the annotated protein sequences against the *cas*-gene repository published earlier [18,33,34,35]. For each detected *cas*-gene, corresponding nucleotide sequences were extracted from the annotation files and searched against the contigs using blastn (options: evalue = 1 × 10^−9^ and qcov_hsp_perc = 95, maximum_bit_score) to find the gene location (sorted by position in the contigs) and orientation (positive or, negative-strand) and define the *cas*-gene loci. The distance from the *cas*-gene loci to the nearby CRISPR loci was also calculated. These data were used to visualize the *cas*-gene loci with nearby CRISPR loci in all *S.* Typhi isolates and compare them among themselves. In the case of detected *cas*-genes of other types of CRISPR-Cas system than I-E, the length of their coding sequences (CDS) was determined and added with their gene name (in superscript) to define an identifier for the CDS. An asterisk (*) was added to all *cas* genes if its CDS had any non-sense mutation and was interrupted prematurely.

The Supplementary Methods provides details on the collection of epidemiological data, generating multilocus sequence typing (MLST), genotype and AMR data, conservation of direct repeats (DR), spacers and their phylogenetic analysis, and finding spacer targets (Doc S1).

## 3. Results

### 3.1. CRISPR Loci of S. Typhi Genomes

**Figure 1:**
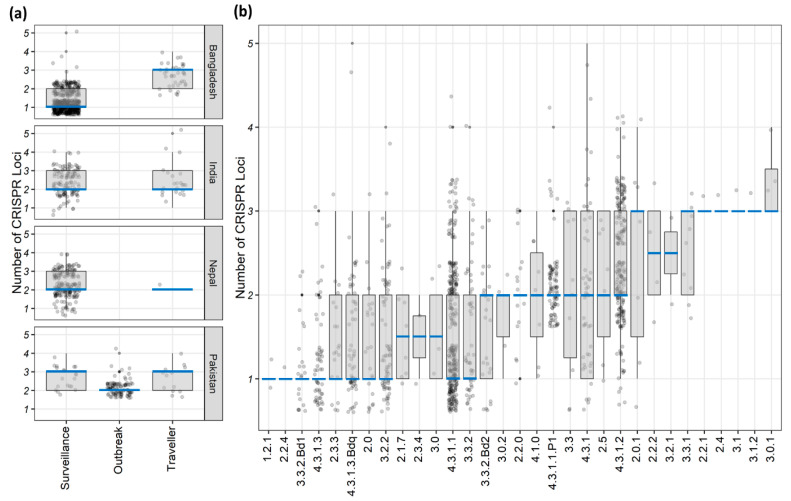
The number of clustered regularly interspaced short palindromic repeats (CRISPR) loci per isolate by (**a**) different countries and study settings, (**b**) different genotypes. In both the boxplots, dots represent the loci number of the isolates, whereas the blue bar indicates the median CRISPR loci number.

A total of 1919 CRISPR loci were detected in the *S.* Typhi genomes analyzed in this study (Table 1). Of them, 55% (1054/1919) were group-A and 45% (865/1919) were group-B CRISPR loci. One to even five CRISPR loci per isolate were detected, but the majority harbored just one (40%) or two (41%) of them (Figure S2). Bangladeshi surveillance isolates showed a lower range of CRISPR loci per strain (1–2; 690/536 vs. 2–3; 1229/523) compared to those from other countries (Figure 1a and Table 1). The extensive drug-resistant (XDR) and non-XDR isolates from the Pakistani outbreak also carried a relatively lower number of CRISPR loci (2–3; 184 loci from 88 XDR isolates of genotype 4.3.1.1.P1 and 26 loci from 12 non-XDR isolates) (Table 1 and Figure 1a). All other isolates across different study settings and countries had a higher number of CRISPR loci (1019 loci from 423 isolates) (Figure 1a). Among the dominant genotypes (with >50 isolates) identified in this study (Table S1), genotype 4.3.1.2 carried the highest average CRISPR loci number (2–3; 509 loci from 213 isolates), whereas the lowest was for 4.3.1.3 (1–2; 70 loci from 55 isolates) (Table 1 and Figure 1b).

The maximum likelihood-based phylogenetic tree (MLT) of all group-A CRISPR loci of *S.* Typhi showed only one primary clade, whereas the MLT was generated with the group-B CRISPR loci had subclades specific to their DR sequences (Figure S3a,b). The MLT of all 1919 group-A and B CRISPR loci showed similar inferences (Figure S4). Surprisingly, 2nd and 3rd group-A CRISPR loci of a Bangladeshi isolate (accession: ERR2663968) were placed outside the primary clade of the MLT (Figure S3a). A blastn analysis confirmed this finding by showing a 100% sequence identity with *Salmonella enterica* serotype Enteritidis and not *S.* Typhi.

**Figure 2:**
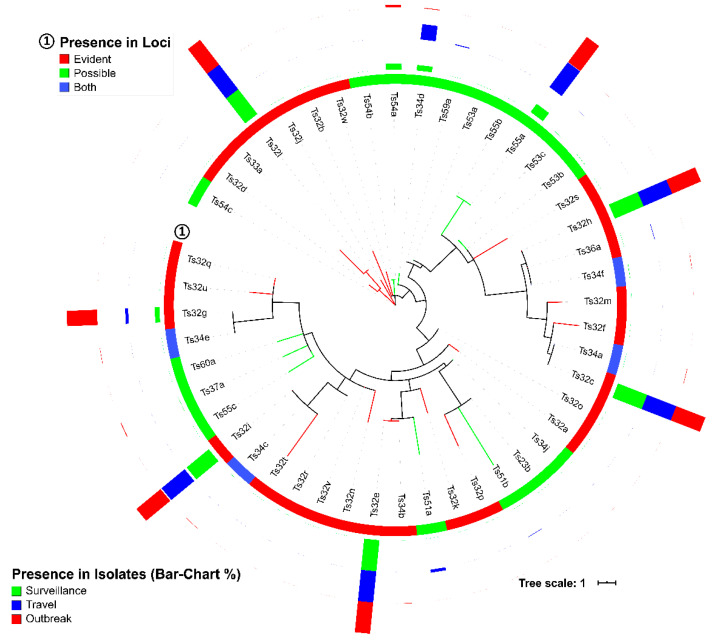
Randomly rooted phylogenetic tree of all spacer sequences (*n* = 47) detected from 1059 *Salmonella enterica* serovar Typhi (*S.* Typhi) isolates in this study (model: K80 + G4). The circle indicates the group of loci and the bars has the percentage of presence in the three different study settings (surveillance, travel, and outbreak).

In the 1919 CRISPR loci, we further identified 15 different DRs, with most having strict specificity toward a certain CRISPR loci group (Table 2 and Figure S3b). Next, 47 unique spacer sequences were detected. Most of the spacers (*n* = 39) showed specificity to either one of the two CRISPR loci groups, except the four spacers named Ts32c, g, h, i, Ts34a, c, e, and, f (Figure 2 and Table 3) (See Figure S1 for details about the identifiers of DR and spacers). Among the highly present spacers, Ts32e and l were only present in group-A loci, whereas Ts55a, Ts54a, and Ts34d showed complete specificity to group-B CRISPR loci (Figure 2 and Table 3). The MLT of all *S.* Typhi spacers did not show any clustering for CRISPR loci group specificity (Figure 2). Instead, group-A and -B CRISPR loci had 7 and 22 different spacer arrangement patterns, respectively, named as a1-7 and b1-22 (Table 4).

**Figure 3:**
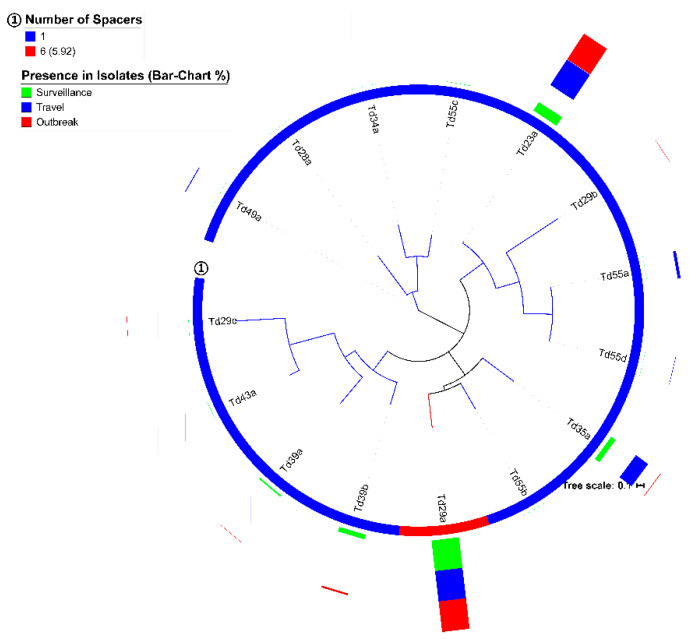
Phylogenetic tree of all direct repeat (DR) sequences (*n* = 15) detected from 1059 *S.* Typhi isolates (randomly rooted; model: JC). The presence of different spacers in different groups of loci is presented in the circle and the average spacer count of each DR is shown on a bar chart (percentage of presence).

All group-A CRISPR loci harbored only one consensus DR, Td29a, that were placed in a subclade with Td35a and Td55b in the MLT of all 15 *S.* Typhi DRs analyzed (Figure 3). CRISPRmap results showed Td29a as a member of superclass-B (SeqFamily-F1) with the M1 structure-motif (Table 5). In contrast, group-B CRISPR loci were dominated by Td23a (53%; 456/885), followed by Td35a (22%; 192/865), Td39b (16%; 139/885), and Td39a (4%; 41/885). Td23a had an M11 structure-motif and belonged to superclass-B, whereas Td39a and Td39b had another structure-motif, M18 (Table 5).

### 3.2. CRISPR Loci of S. Typhi Versus other Salmonella Species

**Figure 4:**
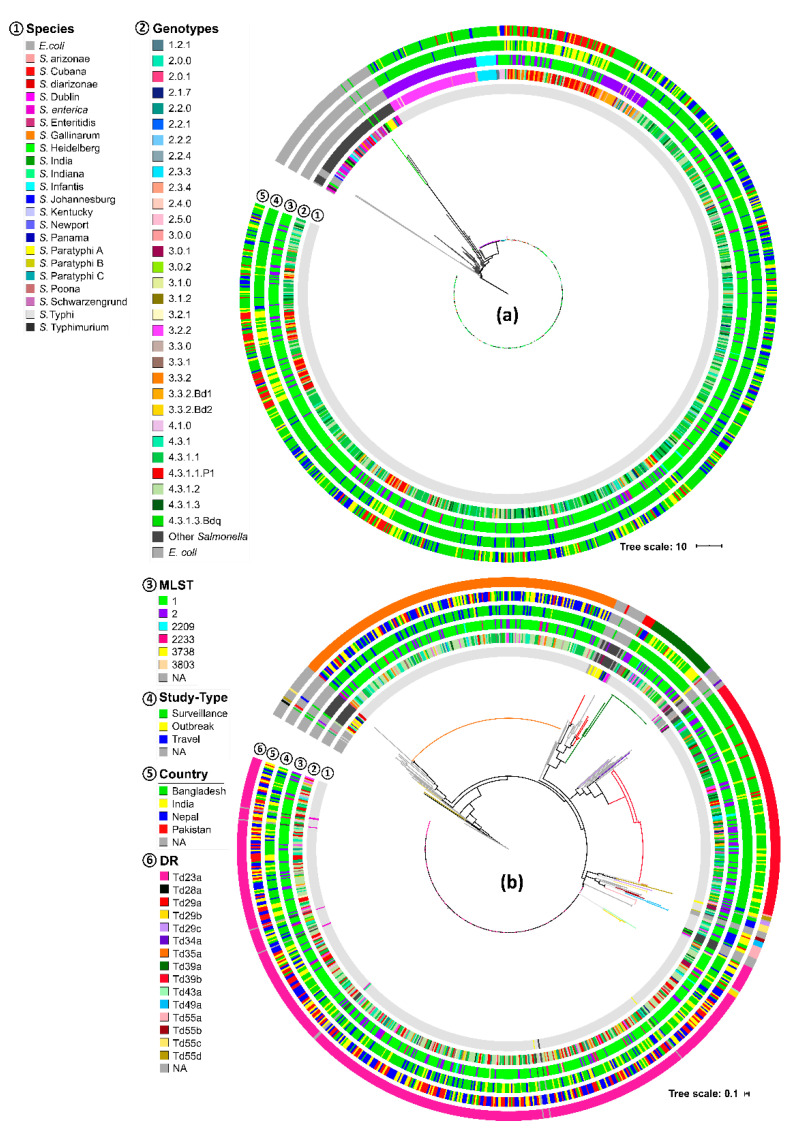
Phylogenetic trees based on CRISPRs that were detected in all *S.* Typhi, other *Salmonella*, and *E. coli* isolate used in this study. The trees are based on all detected (**a**) group-A CRISPRs (Model: K80 + R10) that include 865, 53, and 28 loci, respectively, from *S.* Typhi, *Salmonella* species, and *E. coli*, and (**b**) group-B CRISPRs (model: K80 + G4) (DR: Direct repeats).

The addition of 91 group-A CRISPR loci from other *Salmonella* species (84 loci from 48 isolates of 19 serovars) and *E. coli* (seven loci from six isolates) to the MLT of all *S.* Typhi group-A CRISPR loci (*n* = 1054) revealed the presence of a primary clade (bootstrap 98) specific for *S.* Typhi (Dataset S2 and Figure 4a). The closest neighbor to the clade was *S. enterica* subsp. *enterica* (Figure 4a). This finding obtained further support from the estimated distance (median distance 3.12) calculated from the multiple sequence alignment (MSA) of all 1145 group-A CRISPR loci (Table S2). The lowest intra-species distance (mean 0.07) among group-A CRISPR loci was found for *S.* Typhi (Table S2). Furthermore, the average length of these CRISPR loci was the shortest among all 19 different *Salmonella* serovars studied (after *S. enterica* Dublin; two isolates) (Table S2). Inside the *S.* Typhi serovar specific clade, two small and one large cluster were noticed (100% bootstrap). One small cluster had members of genotype 3.2.2 and the other had 2.3.3 (ST2209), 3.0.0, and 3.2.1 (mostly ST2) from Bangladesh. The larger cluster had all other genotypes identified (Figure 4a and Table S1). No other specific genotype, country-, or MLST-related clustering was identified (Figure 4a).

The MLT of all group-B CRISPR loci did not show exclusive *S.* Typhi clades but re-illustrated the DR sequence specificity (Figure 4b). Only three *S.* Typhi DR sequences (Td35a, Td39a, and Td39b) showed specificity for the serovar, whereas the subclades of other DR sequences were connected to other *Salmonella* species (Figure 4b). MLT of all DR sequences detected in *S.* Typhi, other *Salmonella* spp., and *E. coli* showed striking sequence similarity to the most dominant *S.* Typhi DR, Td29a with five other DR sequences (three from other *Salmonella* serovars and two from *E. coli*), and all were accompanied by high spacer counts (Figure S5a,b).

### 3.3. Spacers and DRs of S. Typhi

**Figure 5:**
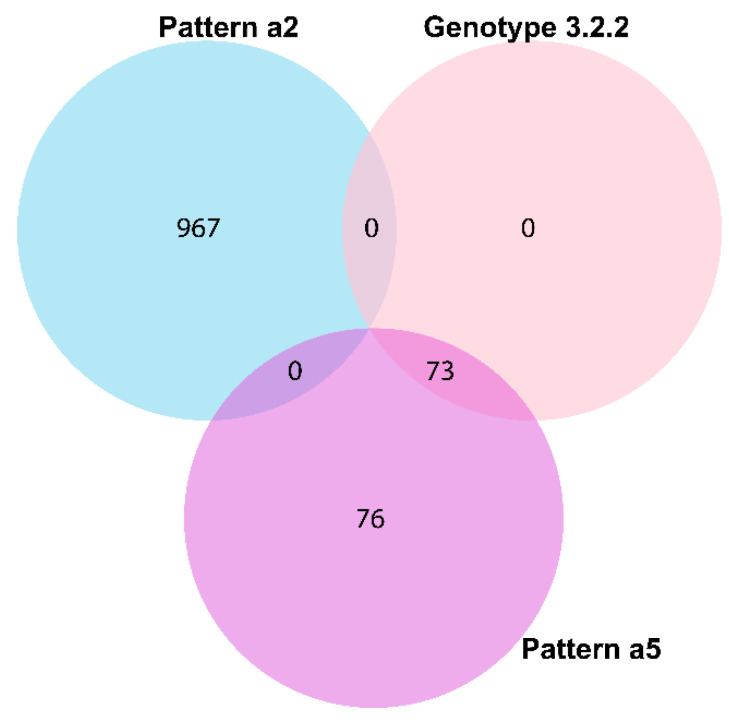
Presence of spacer arrangement patterns of a2 and a5 with a dominant non-multidrug resistance (MDR) genotype 3.2.2.

Among the spacers, Ts32i had ubiquitous presence (*n* = 976) among all study settings, countries, and genotypes, except for the genotype 3.2.2 (*n* = 0; *p* < 0.001). Among others, Ts32c, e, g, h, and l had a high number of presence among *S.* Typhi loci (Table 3). The spacer arrangement pattern, a2, and a5 both presented high specificity (based on presence or absence) to a major non-multidrug resistance (MDR) genotype 3.2.2 (*p* < 0.001) (Figure 5). Among the dominant spacer patterns, a5 was significantly underrepresented in the MDR or XDR group (*p* < 0.001), whereas a2 was present ubiquitously (*p* < 0.001; Figure S6). The same XDR isolates were also dominated by a combined pattern of spacer arrangement, a2–b1 (*n* = 79), however, the pattern was also present in a high number of non-XDR isolates (Figure S6b,c).

A closer look at the presence of different DRs revealed a couple of specific patterns (*n* = 6) as well. The two group-B CRISPR loci specific DRs, Td39a, and Td39b, were more frequently observed among *S.* Typhi isolates obtained from surveillance (*n* = 173) than outbreak (*n* = 7) or travel (*n* = 0) cases (*p* < 0.001) (Table 2 and Figure 3). Two of the dominant DRs, Td23a (*n* = 456) and Td35a (*n* = 192), were almost absent among the Bangladesh surveillance isolates (*n* = 3 and *n* = 6, respectively), whereas the latter DR was only identified in two Pakistan outbreak-related isolates (*p* < 0.001) (Table 2). A few pairs of spacers and DR sequences (Ts34d-Td35a, Ts55a-Td23a, and Ts54a-Td39a/b) also showed specificity to different countries and study settings (*p* < 0.001) (Table S3). Dataset S3 has the sequences of all identified DR and spacers.

### 3.4. Spacer Targets and PAM Identification

**Figure 6:**
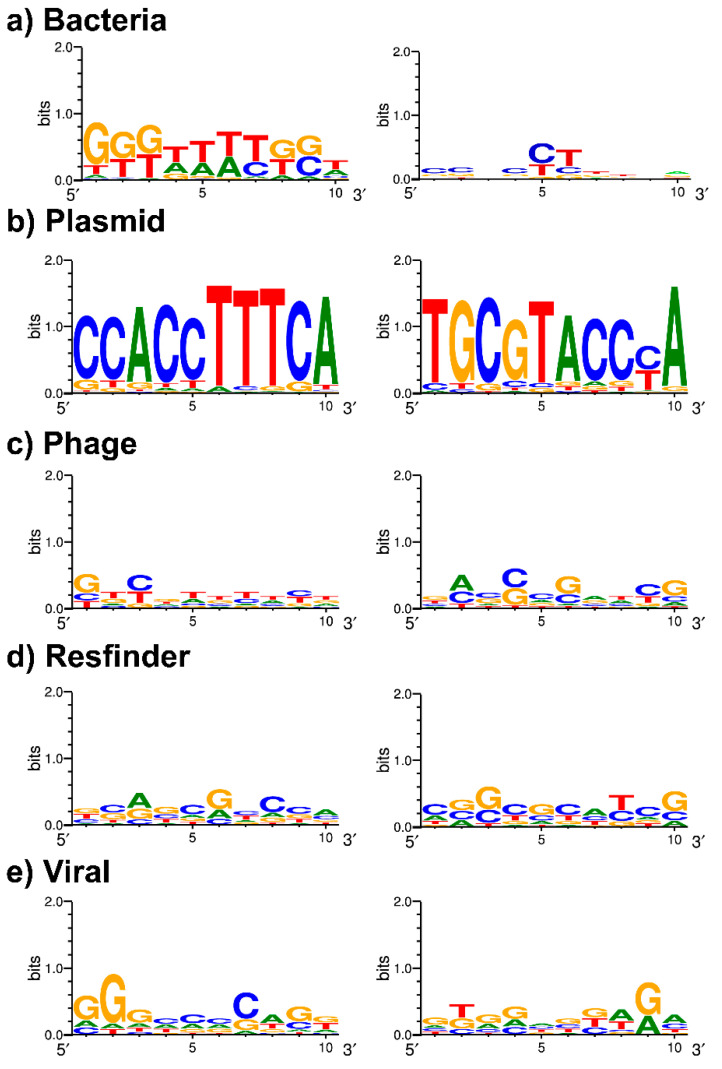
WebLogo results of 10 bp upstream (on left) and downstream (on right) of the spacer-targeted regions based on hits from (**a**) Bacteria, (**b**) Plasmid, (**c**) Phage, (**d**) Resfinder, and (**e**) Viral databases. Conserved regions could be the protospacer adjacent motifs (PAM) for *S.* Typhi.

We thus identified specific spacers, DRs, combined spacer patterns, and DR-spacer pairs of *S.* Typhi that could potentially serve as biomarkers to help identify regional endemicity and AMR amongst others. Only a few spacers harbored 100% (or, nearly 100%) identity with the bacterial, plasmid, phage, viral, and AMR-related sequences (see Doc S1 and Figure S7 for more details and about the databases and filter settings). For all the obtained spacer target hits, the possible PAM sequences (10 bp downstream and upstream of the protospacer) were not conserved, except for the spacers targeting the plasmid sequences (Figure 6a–e). Indeed, the potential PAM regions of plasmid sequences were highly conserved and were marked by the motif TTTCA (upstream) and TGCGT (downstream) (Figure 6b). An almost identical but less conserved motif TTTCT was also observed in the upstream PAM region of the protospacers identified in the phage sequences (Figure 6c). In total, only six different spacers (Ts23b, Ts32a, Ts32g, Ts32i, and Ts32o) harbored protospacers in the phage sequences, all were short in length (23 or 32 bp), mostly present in group-A loci (Table 3 and Table 6). In contrast, five spacers (Ts34j, Ts53a, Ts53b, Ts53c, and Ts59a) that harbored protospacers in the plasmid sequences showed specificity to the group-B CRISPR loci and longer in base-pair length (34, 53 or 59 bp) (Table 3 and Table 6). Each phage-targeting spacer had a different viral target, but none, except Ts32i, targeted a *Salmonella* spp. phage (accession: MK268344.1) (Table 6). Ts32i was present in 91% (974/1059) of the isolates except in genotype 3.2.2 (Table 3). Ts32g was also ubiquitously present (*n* = 1054) and had a target against *Sinorhizobium* phage phiN3 (Table 3 and Table 6). In contrast, all plasmid sequences targeted by the *S.* Typhi spacers were part of only four different “hypothetical” proteins from either the species *Salmonella enterica* or the family *Enterobacteriaceae* (Table 6). None of these proteins showed any hit in the Pfam database (https://pfam.xfam.org/).

### 3.5. Cas Genes

**Figure 7:**
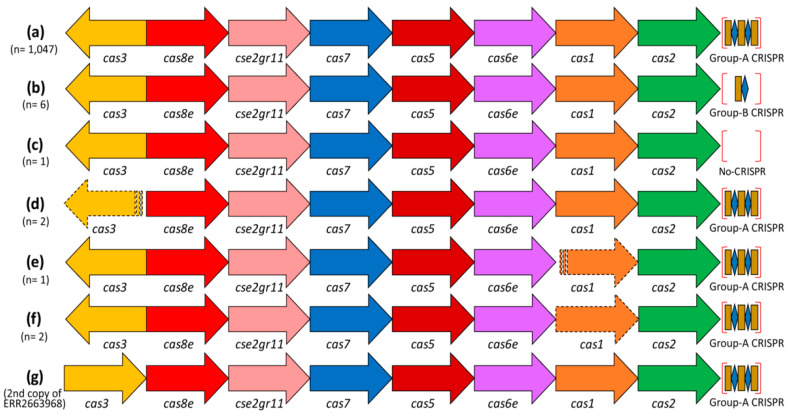
Variation in the arrangement of the type-I-E *cas* genes, their orientations, and the CRISPR loci found in all 1059 *S.* Typhi isolates. Each arrow represents a specific *cas* gene. A stripped arrow indicates segregation of the *cas*-loci into two different contigs and the dashed line of the arrow specifies an interrupted gene. Most of the strains (*n* = 1047) had a group-A CRISPR downstream of the *cas2* gene, six had a group-B locus instead and one strain had none (**a**–**c**). *cas1* and *cas3* genes were split into two different contigs for two and one strains, respectively (**d**,**e**). Another two strains had a non-sense mutation in the *cas1* gene (**e**,**f**). The *cas3* genes in all *cas* gene loci were reversely oriented (in comparison to other *cas* genes), except for the second *cas3* gene identified in ERR2663968. This isolate had two sets of *cas* gene loci with almost the same locus lengths (8453 vs. 8454 bp), gene arrangement, but different sequences (**g**). However, the length of the two sets of the *cas* genes in isolate ERR2663968 was different; *cas1* (918 vs. 921 bp), *cas6e* (705 vs. 651 bp), *cas5* (726 vs. 747 bp), *cse2gr11* (603 vs. 555 bp), *cas8e* (1536 vs. 1557 bp), and *cas3* (2208 vs. 2664 bp).

All 1059 *S.* Typhi isolates had a set of eight *cas*-genes belonging to the type-I-E CRISPR-Cas system. Except for five, all (*n* = 1054) had the same *cas* locus length, gene arrangement, and orientation (Figure 7a–c). Among them, 1047 had a group-A CRISPR locus present, 85-87 bp downstream of the *cas* gene loci, whereas six had a group-B locus at that location and one had none (Figure 7a–c). Five other isolates had a non-sense mutation in their *cas* gene sequence (Figure 7d–f). The isolate (accession: ERR2663968) with two group-A CRISPR loci had two complete sets of *cas* genes (the second set is depicted in Figure 7g). Blastn analysis of the second set of *cas* genes showed >95% sequence identity with other *Salmonella enterica* rather than *S.* Typhi. The *cas* genes of the second set were placed outside the primary clade that contained all other *S.* Typhi *cas* genes in the MLTs. Indeed, this is true for all three MLTs of *cas1*, *cas2*, and *cas3* genes from *S.* Typhi, other *Salmonella* species, and *E. coli* (Figure S8a–c). None of the three MLTs showed any high-bootstrap branching either inside that *S.* Typhi clade, reflecting a high level of conservation and stability of its *cas*-loci (Figure S8). However, the presence of a few other *Salmonella* species inside the *S.* Typhi clade was noticed in the case of *cas2*-MLT (Figure S8b).

In addition to the type-I-E CRISPR-Cas system, all *S.* Typhi isolates had three copies of *DinG*, 2–4 copies of *DEDDh*, and 1–2 copies of *WYL* genes (Figure S9 and Table S4). All three copies of *DinG* and two of the *DEDDh* genes were completely conserved in all 1059 *S.* Typhi isolates (Table S4). Blastn results of a copied variant of the *WYL* gene, *WYL^888^* (*WYL* gene of 888 bp length) showed high sequence identity with a gene that is commonly present on plasmid pK91 (found in *S.* Typhi genotype 4.3.1.3q1), plasmid-2 of the XDR (genotype 4.3.1.1.P1) isolates from Pakistan and pCTXM-2248 of an *E. coli* (accession: MG836696.1) [5,6,7]. Remarkably, it was only present in genotype 4.3.1.3q1 (100%; 56/56) and 4.3.1.1.P1 (XDR, 86/86) isolates (*p* < 0.001) (Figure S9 and Table S4), making it a potential marker for lineage or plasmid identification.

## 4. Discussion

We here show that *S*. Typhi isolates can carry up to five different CRISPR loci and about 19% (203/1059) had three or more CRISPR loci (Figure S2). Although previous studies reported only one or two loci [2,11,12], they analyzed WGS data of a handful of *S.* Typhi isolates, a maximum of 18 genomes by Fabre et al. [11], which could be the reason why others missed the third, fourth, or the fifth loci. However, these isolates carried only one group-A CRISPR locus with a high spacer count, resembles CRISPR1 in the previous nomenclature used by Fabre et al. and it agrees with a few of the previous reports on the CRISPR-Cas system in *S*. Typhi [11,12]. However, nearly 40% (422/1059) of our isolates had only one CRISPR locus and their number was significantly higher among Bangladeshi surveillance isolates, while the Pakistani outbreak isolates had a relatively lower average loci number (*p* < 0.001; Figure 1a and Table 1). Local and highly clonal *S.* Typhi lineages have been reported from both countries [5,6] and none of these lineages had higher average numbers of CRISPR loci (Figure 1b). Hence, clonality could be a contributing factor for the lower number of CRISPR loci identified in these isolates.

Haplotype specificity of the *S.* Typhi spacer arrangement patterns has been described [11]. We could not confirm those associations [11], primarily, because we were unable to identify the same spacers, except one, Ts32v (match 31/32 bp of a spacer from CRISPR2 described by Fabre et al. [11]). However, our study revealed multiple spacers (Ts32g, Ts32h, and Ts32i), spacer arrangement patterns (a2 and a5), DRs (Td23a, Td35a, and Td39a-b), and DR-spacer pairing patterns (Ts34d-Td35a, Ts55a-Td23a, and Ts54a-Td39a/b) specific to different AMR, country, genotype or surveillance, travel, and outbreak characteristics (Figure 2, Figure 3 and Figure 5, Table 2 and Table 4, Figure S6, Table S3, and Dataset S1). The identified spacer, DR, and DR-spacer patterns could, therefore, be further exploited by CRISPR-based diagnostic platforms like SHERLOCK or DETECTR for clinically relevant samples [36,37] to identify AMR among endemic isolates that are spreading in and beyond South Asian countries [29,38].

The spacer sequences of *S.* Typhi showed remarkable conservation, and only 47 unique spacers were detected in 1919 CRISPRs identified in the genomes of 1059 *S*. Typhi isolates (Table 3 and Dataset S3). Many spacers in group-A loci (Ts32c, e, g, h, i, and l) were almost universally present in all *S.* Typhi isolates, whereas specific spacers (Ts55a, Ts54a, Ts34d) showed high numbers of presence in group-B loci (Figure 2 and Table 3). Reports on CRISPRs identified in other pathogens described a higher number of unique spacers, i.e., 2823 spacers from 669 *Pseudomonas aeruginosa* and 745 from 100 *E. coli* isolates [26,39]. In our study, 48 other *Salmonella* (19 different serovars) and six *E. coli* isolates showed 857 unique spacers from 136 CRISPR loci and 118 unique spacers from 35 loci identified in their genome, respectively (*data not shown*). However, a study of 400 *Salmonella enterica* isolates of four serovars (Enteritidis, Typhimurium, Newport, and Heidelberg) reported 179 unique spacers [21]. A lower number of unique spacers have also been reported for pathogens like *Campylobacter jejuni*, *Neisseria meningitidis*, *Pasteurella multocida*, *Streptococcus agalactiae,* and *Shigella* spp. [40,41]. Such conservative nature of *S.* Typhi spacers could be due to host-restriction of *S*. Typhi.

It is now well established that spacers are likely to share complementarity with a target sequence (protospacer) in foreign DNA. The *S.* Typhi CRISPRs have been studied before, but the PAM sequence was yet to be defined. In our work, we report for the first time a possible PAM sequence, TTTCA/T. Although this PAM is based on the protospacers of only nine different spacers (Table 6), the nearly universal presence of two phage-targeting spacers, Ts32g (*n* = 1054) and Ts32i (*n* = 976), make this PAM motif more plausible. Besides that, Ts32i also targets a *Salmonella* phage suggestive for a functional CRISPR-Cas-related viral immunity system to protect the *S.* Typhi genome against bacteriophages.

Furthermore, the differentiation between the spacers or DRs of group-A and -B CRISPR loci were evident in our work. Very few spacers (*n* = 8) and DRs (*n* = 1) were present in both groups and considering the spacer targets, the *S*. Typhi group-A CRISPR loci seem more associated with phage defense, whereas group-B CRISPR loci potentially play a role in the defense against plasmids (Table 3 and Table 6). This is not a common finding since the reports of defense mechanisms in other bacterial species against phages and plasmids are mainly linked to group-A CRISPR loci [42,43,44].

Similar to the previous reports [11,12,13], the CRISPR-Cas system identified in our study belongs all to the type I-E category in the case of S. Typhi. Among the identified *cas* genes, very few (*n* = 5) had an incomplete reading frame (Figure 7), which could be caused by non-sense mutations or sequencing errors. However, all *cas* gene loci were detected near a group-A locus, except six, where a group-B locus was present instead (Figure 7b). Thus, most of the group-B loci can be called “orphan” loci. According to the CRISPRCasFinder tool, CRISPR loci with low evidence score (which we termed group-B loci) might be false-positive, but some of the CRISPR arrays can be real. Indeed, the CRISPRCasFinder tool was specifically designed to identify these types of CRISPR loci so they could be functionally studied [32]. To our knowledge, orphan loci have never been reported for *S.* Typhi before. However, as identified in other prokaryotes, they can exist and even be functional without nearby *cas*-gene loci [15,16,18,32,45,46].

We also identified three different *cas* genes of other types of CRISPR-Cas system, i.e., *DinG*, *DEDDh*, and *WYL* (Figure S9 and Table S4). Although the presence of the *DinG* family helicase gene suggests an existing type-IV-A CRISPR-Cas system [33], no other *cas*-genes of that system were found. No CRISPR loci were present on the same contigs either, but that is not uncommon for this type of system [16,18]. The type IV-A system is considered as a degraded derivative of class 1 CRISPR-Cas system, hypothesized to be originating from combinations of mobile genetic elements [16,18,47]. The presence of multiple copies of the *WYL* gene (part of the type-I system) among the *S*. Typhi isolates in our study was interesting, as two copies of this gene, *WYL^693^* and *WYL^888^,* had a difference in origin and presence. The former had a chromosomal match, whereas the latter was probably plasmid-borne (Table S4). *WYL^888^* matched the plasmid sequences of genotype 4.3.1.3q1 (Bdq lineage) and 4.3.1.1.P1 (XDR lineage) [5,6,7], making it a potential biomarker for these resistance lineages. However, the role of *WYL^888^* on these plasmids remains to be elucidated. Remarkably, both the *S.* Typhi lineages completely lacked a copy of the *DEDDh^558^* gene (Table S4). Proteins containing the WYL domain are not uncommon in bacteria and have been reported to regulate transcription of the CRISPR-Cas systems [48]. The *DEDDh* gene, on the other hand, has defined exonuclease activity and can fuse with *cas1* and *cas2* genes to exert such function [49,50]. The presence of multiple DEDDh domains in *S.* Typhi genomes may indicate a compensatory role for the shorter *cas3* gene (compared to other *Salmonella* species, *data not shown*), which also functions as an exonuclease.

## 5. Conclusions

In conclusion, this study is the first large-scale bioinformatic investigation of the *S.* Typhi CRISPR-Cas system identified in the genomes obtained from isolates studied in different backgrounds and four endemic countries. Our results reveal unique conservation and clonality of the *S*. Typhi type I-E CRISPR-Cas system, specifically the *cas*-genes. Despite the clonality of this system, variations were identified in the type I-E CRISPR-Cas system of *S*. Typhi that significantly associated with AMR status, genotypes, demographic origin, and endemic isolates currently circulating in the south Asian countries. Although no AMR-gene targeting spacers were found, spacers targeting the AMR-containing plasmids were identified. This indicates a lack of a direct CRISPR-regulated pathway, rather regulating the AMR-gene acquisition or elimination via controlling the entry of plasmids. Finally, a possible *S.* Typhi PAM sequence, TTTCA/T, was defined in this study. Our findings lay a foundation for new genetic and biochemical experiments to dissect the CRISPR-Cas system of *S.* Typhi further and gain mechanistic insights into its molecular function. Overall, the strong correlations of variations identified in the system with AMR and demographic data of the endemic isolates from south Asian countries should be investigated further with keeping the development of rapid and inexpensive diagnostic tests as a target.

## Figures and Tables

**Table 1:** The number of average CRISPR loci (range) by country and genotype/lineages (all loci numbers are given in “*range (median)*” format). By country, Bangladesh-surveillance had the lowest range of CRISPR loci number (1–2). By genotype—4.3.1.1, 4.3.1.3, 4.3.1.3q1, 2.0, 2.3.3, 3.2.2, 3.3.2 and 3.3.2.Bd1 had one locus per isolate (median).

Different Datapoints			Study Type									
			Surveillance				Outbreak	Travel				Total
Country			Bangladesh	India	Nepal	Pakistan	Pakistan	Bangladesh	India	Nepal	Pakistan	-
Total number of Isolates			536	131	198	20	100	38	22	1	13	1059
Total number of CRISPR loci			690	317	457	53	210	102	54	2	34	1919
Range of CRISPR loci			1–2	2–3	2–3	2–3	2–3	2–3	2–3	2-3	2-3	1–2
Number of isolates and average CRISPR loci number by genotypes (genotypes with total ≥10 isolates)	4.3.1	No. of Isolates	15	11	6	4	5	-	5	-	4	50
		Loci number	1–2 (1)	2–3 (2)	2–3 (2)	3 (3)	2 (2)	-	3–4 (3)	-	2–3 (2.5)	2–3 (2)
	4.3.1.1	No. of Isolates	223	24	15	7	4	19	2	-	4	298
		Loci number	1–2 (1)	2 (2)	2–3 (3)	2–3 (3)	2 (2)	2–3 (3)	2 (2)	-	3 (3)	1–2 (1)
	4.3.1.1.P1	No. of Isolates	-	-	-	-	88	-	-	-	-	88
		Loci number	-	-	-	-	2–3 (2)	-	-	-	-	2–3 (2)
	4.3.1.2	No. of Isolates	4	59	133	1	2	1	11	1	1	213
		Loci number	1–2 (1)	2–3 (3)	2–3 (2)	2 (2)	2–3 (2.5)	2 (2)	2–3 (2)	2 (2)	2 (2)	2–3 (2)
	4.3.1.3	No. of Isolates	53	-	-	-	-	2	-	-	-	55
		Loci number	1–2 (1)	-	-	-	-	3 (3)	-	-	-	1–2 (1)
	4.3.1.3q1	No. of Isolates	55	-	-	-	-	1	-	-	-	56
		Loci number	1–2 (1)	-	-	-	-	3 (3)	-	-	-	1–2 (1)
	2.0.0	No. of Isolates	18	1	1	3	-	-	-	-	-	23
		Loci number	1–2 (1)	2 (2)	2 (2)	2–3 (2)	-	-	-	-	-	1–2 (1)
	2.2.0	No. of Isolates	3	1	10	-	-	-	-	-	1	15
		Loci number	1–2 (1)	2 (2)	2 (2)	-	-	-	-	-	3 (3)	2 (2)
	2.3.3	No. of Isolates	18	-	-	-	-	2	-	-	-	20
		Loci number	1–2 (1)	-	-	-	-	2–3 (2.5)	-	-	-	1–2 (1)
	3.2.2	No. of Isolates	61	2	6	1	-	2	-	-	1	73
		Loci number	1–2 (1)	2–3 (2.5)	2–3 (1)	3 (3)	-	3 (3)	-	-	3 (3)	1–2 (1)
	3.3	No. of Isolates	1	4	3	1	-	-	-	-	1	10
		Loci number	3 (3)	2–3 (3)	1 (1)	2 (2)	-	-	-	-	2 (2)	2-3 (2)
	3.3.2	No. of Isolates	32	1	16	-	-	1	-	-	-	50
		Loci number	1–2 (1)	4 (4)	2-3 (2)	-	-	3 (3)	-	-	-	1–2 (1)
	3.3.2.Bd1	No. of Isolates	19	-	-	-	-	2	-	-	-	21
		Loci number	1–2 (1)	-	-	-	-	2 (2)	-	-	-	1–2 (1)
	3.3.2.Bd2	No. of Isolates	17	-	-	-	-	7	-	-	-	24
		Loci number	1–2 (1)	-	-	-	-	2–3 (2)	-	-	-	1–2 (2)

**Table 2:** Presence of different *S*. Typhi DR sequences in different groups of loci by study type and country.

DR UniqueID	All		Surveillance (Bangladesh)		Surveillance (India, Nepal, Pakistan)		Surveillance (All)		Travel		Outbreak	
	Group-A	Group-B	Group-A	Group-B	Group-A	Group-B	Group-A	Group-B	Group-A	Group-B	Group-A	Group-B
Td23a		456		3		282		285		72		99
Td28a		1		1				1				
Td29a	1054	7	535	3	345	4	880	7	74		100	
Td29b		3		2				2				1
Td29c		3		2				2				1
Td34a		1		1				1				
Td35a		192		6		148		154		36		2
Td39a		41		19		21		40				1
Td39b		139		117		16		133				6
Td43a		1		1				1				
Td49a		3				1		1		2		
Td55a		8				1		1		7		
Td55b		2				2		2				
Td55c		5				5		5				
Td55d		3				2		2		1		

**Table 3:** Presence of different *S.* Typhi spacer sequences in different groups of loci by study type and country.

Spacer UniqueID	All		Surveillance (Bangladesh)		Surveillance (India, Nepal, Pakistan)		Surveillance (All)		Travel		Outbreak	
	Group-A	Group-B	Group-A	Group-B	Group-A	Group-B	Group-A	Group-B	Group-A	Group-B	Group-A	Group-B
Ts23b		1		1				1				
Ts32a	1		1				1					
Ts32b	1		1				1					
Ts32c	1050	3	531		345	3	876	3	74		100	
Ts32d	1		1				1					
Ts32e	1050		533		345		876		74		100	
Ts32f	1		1				1					
Ts32g	1052	2	533	2	345		878	2	74		100	
Ts32h	1052	7	533	3	345	4	878	7	74		100	
Ts32i	974	2	471	2	332		803	2	71		100	
Ts32j	2		2				2					
Ts32k	1		1				1					
Ts32l	1047		533		342		875		73		99	
Ts32m	1		1				1					
Ts32n	1		1				1					
Ts32o	1		1				1					
Ts32p	1		1				1					
Ts32q	1		1				1					
Ts32r	1		1				1					
Ts32s	1		1				1					
Ts32t	1		1				1					
Ts32u	1		1				1					
Ts32v	1		1				1					
Ts32w	1		1				1					
Ts33a	1		1				1					
Ts34a	1	3				3		3			1	
Ts34b	1										1	
Ts34c	1	2		2				2			1	
Ts34d		192		6		148		154		36		2
Ts34e	1	2		2				2			1	
Ts34f	1	7		3		4		7			1	
Ts34j		1		1				1				
Ts36a	4				3		3		1			
Ts37a		6		4				4				2
Ts51a		8				1		1		7		
Ts51b		3				2		2		1		
Ts53a		2				2		2				
Ts53b		4				4		4				
Ts53c		1				1		1				
Ts54a		178		134		37		171				7
Ts54b		1		1				1				
Ts54c		1		1				1				
Ts55a		454		3		280		283		72		99
Ts55b		1				1		1				
Ts55c		1				1		1				
Ts59a		3				1		1		2		
Ts60a		1		1				1				

**Table 4:** Different arrangement patterns of the spacers in *S.* Typhi isolates in this study. Each arrangement was considered as an “array pattern” and labeled after their loci type with a number (started with “a” for group-A loci, e.g., a1, a2, etc. and “b” for group-B loci, e.g., b1, b2, etc.).

Loci Group	Pattern Names	Loci Length (bp)	DR	Spacer Arrangements
Group-A	a1	517	Td29a	Ts32d, Ts32a, Ts32k, Ts32p, Ts32s, Ts32q, Ts32u, Ts32t
	a2 *	395, 421, 447, 499	Td29a	Ts32h, Ts32c, Ts32l, Ts32e, Ts32i, Ts32g
	a3	579	Td29a	Ts32m, Ts32o, Ts32r, Ts32b, Ts33a, Ts32w, Ts32n, Ts32f, Ts32v
	a4 *	332, 356	Td29a	Ts32h, Ts32c, Ts32e, Ts32i, Ts32g
	a5	360	Td29a	Ts32g, Ts32e, Ts32l, Ts32c, Ts32h
	a6	421	Td29a	Ts32g, Ts32i, Ts32e, Ts32l, Ts32j, Ts32h
	a7 *	273, 299	Td29a	Ts32g, Ts32l, Ts32c, Ts32h
Group-B	b1	102	Td23a	Ts55a
	b2	102	Td23a	Ts55b
	b3	102	Td23a	Ts55c
	b4	89	Td29a	Ts32h
	b5	80	Td28a	Ts23b
	b6	96	Td29b	Ts37a
	b7	96	Td29c	Ts37a
	b8	129	Td34a	Ts60a
	b9	105	Td35a	Ts34d
	b10	133	Td39a	Ts54a
	b11	133	Td39a	Ts54c
	b12	133	Td39b	Ts54a
	b13	133	Td39b	Ts54b
	b14	121	Td43a	Ts34j
	b15	158	Td49a	Ts59a
	b16	162	Td55a	Ts51a
	b17	164	Td55b	Ts53a
	b18	164	Td55c	Ts53b
	b19	164	Td55c	Ts53c
	b20	162	Td55d	Ts51b
	b21	150	Td29a	Ts32c, Ts32h
	b22	211	Td29a	Ts32g, Ts32i, Ts32h

* Multiple probable deletion events were detected, which caused the variation in the length. In the pattern a2, most loci had 421 bp length (*n* = 734), followed by 395 (*n* = 215), 447 (*n* = 16) and 499 bp (*n* = 2).

**Table 5:** CRISPRmap results of all the direct repeat (DR) sequences (*n* = 15) detected from 1059 *S.* Typhi isolates.

DR Unique ID	Sequence	Presence in Number of Isolates	Presence in Group-B Loci	Length of Group-B Loci	CRISPRmap Findings				
					CRISPRmap ID	Structural Motif	Sequence Family	Sub-Type	Superclass
Td23a	GCTTCAGTGGCGAACGTCGTGAA	456	456	101		motif 11	-	-	D
Td28a	TTTTGATGTACTTTTGATGTAATTCTGT	1	1	79		-	-		-
Td29a	GTGTTCCCCGCGCCAGCGGGGATAAACCG	1059	7	88, 149, 210	Crod_A_G_10_M1_F1	motif 1	family 1	I-E	B
Td29b	GTGGGTGGACAGGCTGGACAAAGTGGACA	3	3	95		-	-		-
Td29c	TGTCCACTTTGTCCAGTCTGTCCACCCAC	3	3	95		-	-		-
Td34a	TATATTGGGTGATTACAACTCGTTGAAAAATAAG	1	1	128		-	-		F
Td35a	GTAGACCCTGATCCAGTAGACCCGGTTATCCCTGA	192	192	104		-	-		-
Td39a	CCAGCTTCTGAGCTGCGAATGCGCTGCTGACAGCGGTAC	41	41	132		motif 18	-		-
Td39b	GTACCGCTGTCAGCAGCGCATTCGCAACTCAGAAGCTGG	139	139	132		motif 18	-		-
Td43a	TGCGTACCCATCCACCTTTCAGTGCGTACCCATCCACCTTTCA	1	1	120		motif 11	-		-

**Table 6:** Detected targets of *S.* Typhi spacer against the plasmid and phage database. The target-finding algorithm is illustrated in Figure S7.

Database	Spacer Name	Genbank Accession	Description	Size
Phage	Ts32a	KY006853.1	*Erythrobacter* phage vB_EliS_R6L	65,675 bp
Phage	Ts32g	KR052482.1	*Sinorhizobium* phage phiN3	206,713 bp
Phage	Ts32i	MK268344.1	*Salmonella* phage Munch	350,103 bp
Phage	Ts32o	KY045851.1	*Pseudoalteromonas* phage C5a	35,209 bp
Phage	Ts32o	MG592431.1	*Vibrio* phage 1.049.O._10N.286.54.B5	45,021 bp (partial genome)
Phage	Ts32o	MG592432.1	*Vibrio* phage 1.050.O._10N.286.48.A6	45,285 bp (partial genome)
Plasmid	Ts34j	WP_128853136.1	MULTISPECIES: hypothetical protein [*Enterobacteriaceae*]	72 aa
Plasmid	Ts53a	WP_053521168.1	hypothetical protein [*Salmonella enterica*]	62 aa
Plasmid	Ts53a	WP_071785737.1	hypothetical protein [*Salmonella enterica*]	59 aa
Plasmid	Ts53a	WP_071790422.1	hypothetical protein [*Salmonella enterica*]	76 aa
Plasmid	Ts53b	WP_053521168.1	hypothetical protein [*Salmonella enterica*]	62 aa
Plasmid	Ts53b	WP_071785737.1	hypothetical protein [*Salmonella enterica*]	59 aa
Plasmid	Ts53c	WP_053521168.1	hypothetical protein [*Salmonella enterica*]	62 aa
Plasmid	Ts53c	WP_071785737.1	hypothetical protein [*Salmonella enterica*]	59 aa
Plasmid	Ts59a	WP_053521168.1	hypothetical protein [*Salmonella enterica*]	62 aa
Plasmid	Ts59a	WP_071785737.1	hypothetical protein [*Salmonella enterica*]	59 aa

## Supplementary Materials

The following are available online at https://www.mdpi.com/2073-4425/11/11/1365/s1, Dataset S1: Complete data of all *S.* Typhi isolates used in this study, including the ENA accessions, metadata from the source articles, and the results we generated for each isolate like- MLST, genotype, number of CRISPR loci, spacer patterns, and presence of newly described *cas*-genes. Dataset S2: Accession numbers of 48 other *Salmonella* (19 different serovars, excluding Typhi) and six *E. coli* isolates. Dataset S3: Fasta sequence file of all *S.* Typhi direct repeats (DR) and spacers identified in this study. Doc S1: Describes the additional parts of the Method and Material section. Figure S1: An explanation of the identifier we generated for each unique direct repeat (DR) and spacer sequence in this study. Figure S2: Number of CRISPR loci present per *S.* Typhi isolate. Maximum five different loci were found. Figure S3: Phylogenetic trees of CRISPRs detected in all 1059 *S.* Typhi isolates in this study. The trees are based on all detected (a) group-A CRISPR loci (model: TPM2+F+G4) and (b) group-B CRISPR loci (model: K80+G4) (MDR: Multidrug resistance, XDR: Extensively drug resistant, CIP: ciprofloxacin, DR: Direct repeats.). Figure S4: Phylogenetic trees of all 1919 group-A and B CRISPR loci detected from 1059 *S.* Typhi isolates in this study (model: TVM+FC) (MDR: Multidrug resistance, XDR: Extensively drug resistant, CIP: ciprofloxacin). Figure S5: (a) Randomly rooted phylogenetic tree of all direct repeat (DR) sequences (*n* = 69) detected in 1059 *S.* Typhi, 48 different *Salmonella* (19 serovars), and six *E. coli* isolates in this study (model: JC+G4m). The most common *S.* Typhi DR sequence, Td29a, and its closely related DRs from other species are highlighted in yellow. (b) Multiple sequence alignment of closely related DRs from other *Salmonella* and *E. coli* of the most dominant *S.* Typhi DR, Td29a. Figure S6. Presence of different spacer arrangements (array patterns) among MDR and XDR isolates. (a) all array patterns of group-A CRISPRs, (b) all array patterns of group-B CRISPRs, c) all array patterns of all combined (group-A and B) CRISPR loci. Figure S7: Flow-chart showing the details of spacer-target finding algorithm. Figure S8: Phylogenetic trees of all *S.* Typhi, other *Salmonella* and *E. coli* isolates in this study, based on (a) *cas1* (model: LG+G4), (b) *cas2* (model: LG+G4), and (c) *cas3* (model: PROTGAMMAVTF) gene sequences. Figure S9: Presence of the identified *cas* genes of other types of CRISPR-Cas system than type-I-E in all 1059 isolates. Each arrow represents a specific *cas* gene. A stripped arrow indicates the location of the *cas*-genes into two different contigs and the dashed line of the arrow specifies an interrupted gene. Except for the *DinG^*924^* and *DinG^*1212^* variants of the *DinG* gene, none were located on the same contig. Table S1: Different *S.* Typhi genotypes identified in this study. Table S2: Estimated distance within and between different *Salmonella* serovars including *S.* Typhi based on multiple sequence alignment of all group-A CRISPR loci sequences. Table S3: Presence of multiple DR-spacer pairs in different genotypes, countries, and study settings. Table S4: Details on different copies of *DinG*, *DEDDh,* and *WYL* genes. The length of the different copies of the genes was determined and added with their gene name (in superscript) to define an identifier for the coding sequence (CDS). An asterisk (*) was added to all *cas* genes of an isolate if the CDS had any non-sense mutation and interrupted prematurely.
