# Supplementary material for: CRISPR-Cas Diversity in Clinical Salmonella enterica Serovar Typhi Isolates from South Asian Countries
Source: Genes (Basel). 2020 Nov 18;11(11):1365. doi: 10.3390/genes11111365 (PMC7698835; doi:10.3390/genes11111365)
Supplement: Supplementary file 1 [file genes-11-01365-s001.zip › Doc S1_Final.docx]

**Supplementary Methods**

*Collecting the epidemiological data*

We extracted all metadata (for each *S.* Typhi isolate) available from the five source articles wherein these 1,059 isolates were described [5,6,28-30]. However, only country, year, and genotype data were found for all 1,059 isolates. Age of the patients and study-settings were available from Tanmoy AM *et* *al*., 2018 (Bangladesh), and Britto CD *et* *al*., 2018 (Nepal) [5,28].

*Generating MLST, genotype, and AMR data*

Except for the isolates from Tanmoy AM *et* *al*., 2018 (Bangladesh) ([5](#_ENREF_5)), none had the MLST data available. So, the Achtman 7-gene MLST scheme was followed on EnteroBase (<http://enterobase.warwick.ac.uk/species/senterica/search_strains?query=st_search>) to define the Sequence Type (ST) of the remaining 523 isolates (**Dataset S1**). Although most of the isolates had their genotype and lineage data available, those were generated using an old version of the genotyphi script (<https://github.com/katholt/genotyphi>) [28,30]. Moreover, strains from Wong VK *et* *al*., 2015, and 2016 [29,30] were not screened for the presence of the recently proposed genotypes, i.e. 4.3.1.1.P1, 3.3.2.Bd1 and 3.3.2.Bd2 [29,30,51]. Tanmoy AM *et* *al*., 2018 [5] also described a new lineage of genotype 4.3.1 (lineage Bd), which was later renamed as 4.3.1.3 [5,51]. The same article also described a highly ciprofloxacin-resistant sub-lineage of genotype 4.3.1.3 named Bdq [5], thus we renamed this sub-lineage as genotype 4.3.1.3q1.

To re-generate the *S.* Typhi genotype data, the assembled contigs were mapped against the *S.* Typhi CT18 reference genome using bwa-mem (default options), samtools (view -bS, sort, index, mpileup -BAuf), and bcftools (call -m) [52-56]. Next, the mapped data (vcf file) was screened with the genotyphi script (updated version from June 7th, 2019). However, the script does not include the detection strategy of genotype 4.3.1.3q1. So, we used a customized python script for this purpose (named “DetectBdq.py”). This script is available here (<https://github.com/arif-tanmoy/DetectBdq>).

Antimicrobial resistance (AMR) data was gathered from the source articles, focusing on the availability of MDR data, but found incomplete or unavailable for several cases. Thus, to normalize the data, a new AMR profile was generated for each isolate based on the presence of the resistance genes or mutations using pathogenwatch (<https://pathogen.watch/>) (**Dataset S1**). Any isolate with resistance to broad-spectrum cephalosporin was considered as ceftriaxone resistance.

*Conservation of DR sequences, spacer targets, and analysis with PAM*

Conservation of all DR sequences was checked using CRISPRmap webtool [57]. If matched with the database, motifs, family, and superfamily data were collected. To detect the spacer targets, five different databases were used: Bacteria, plasmid, and viral databases were downloaded from NCBI-RefSeq (FTP release 93) (ftp://ftp.ncbi.nlm.nih. gov/refseq/release/) and Phage database was from NCBI-Genbank (FTP release 230) (<ftp://ftp.ncbi.nlm.nih.gov/genbank/>) (downloaded on 31^st^ March 2019). AMR gene database was taken from Resfinder v3.1 (<https://cge.cbs.dtu.dk/services/ResFinder/>) [58]. A complete algorithm to find a target for the spacers is described in **Figure S7**. In short, four different parameters were used to filter the primary hits from each database, i.e. i) sequence identity, ii) coverage, iii) mismatch and iv) gaps. The threshold values of these parameters were different for each database (**see Figure S7** for details). If any protein-coding sequence was identified as a spacer target, the amino-acid sequence of the protein was extracted from the NCBI-Protein database (https://www.ncbi. nlm.nih.gov/protein) and matched against the Pfam database [59]. For all spacer targets, 10 bp sequences were extracted from downstream and upstream regions to search for possible protospacer adjacent motifs (PAM). All possible PAM sequences from each database were aligned individually and checked for conservation using WebLogo (https://weblogo. berkeley.edu/logo.cgi) [60].

*Phylogenetic analysis and estimating distance*

To construct phylogenetic trees of the *cas1, cas2,* and *cas3* genes, the same prokka-annotated amino acid files were used to extract the genes of interest [35]. Unique direct repeat (DR) and spacer sequences were used for individual phylogenetic construction as well to observe their association. Phylogeny of *cas* genes and DR had two sample sets, i) All *S.* Typhi and, ii) All *S.* Typhi, *Salmonella,* and *E. coli*. Any *cas* gene with non-sense mutations (prematurely ended) was excluded from the analysis. The sample-set for spacer phylogeny only included all *S.* Typhi spacer sequences. For the phylogeny of group-A and B CRISPR loci, we took all CRISPR sequences from CRISPRFinder output. All CRISPR (group-A and B individually) phylogeny were run on two different sample sets, namely- i) All *S.* Typhi (n=1,059) and ii) All *S.* Typhi, *Salmonella,* and *E. coli* (n= 1,113). All group-A and B CRISPR loci of *S.* Typhi (n=1,919) were again considered together for a combined phylogeny.

Muscle v3.8.31 was used to generate multiple sequence alignments (MSA) of all sample sets and iqtree v1.6.8 to find the best-fit substitution model [61,62]. Following the best-fit model, a maximum-likelihood (ML) based phylogenetic tree (MLT) was built for each sample set using RAxML-NG (100 bootstraps) and visualized with iTol v4 [63,64]. Names of the substitution models of each MLT are given in the Figure legend.

To root all CRISPR and Cas MLTs, sequences of *E. coli* str. K-12 (accession: NC_000913.3) was included as an outgroup. MLTs of DR and spacer sequences were either left unrooted or rooted randomly. The MSA of group-A CRISPR loci was used to estimate the distance between different groups of *Salmonella* serovars using DiStats (options: --nosubspecies) [65].

*Analyzing Metadata*

All *S.* Typhi data (genome, CRISPR, AMR, epidemiological, genotype, MLST-types) were merged in a single file (**Dataset S1**) and analyzed using Stata SE v13.0. (StataCorp, TX, USA). A Chi-square test was used to determine the significance of specificity or absence of any element (spacer, DR, or spacer arrangement patterns). Graphs and Venn diagrams were generated using R.

**References**

5. Tanmoy, A.M.; Westeel, E.; De Bruyne, K.; Goris, J.; Rajoharison, A.; Sajib, M.S.I.; van Belkum, A.; Saha, S.K.; Komurian-Pradel, F.; Endtz, H.P. *Salmonella enterica* serovar Typhi in Bangladesh: Exploration of Genomic Diversity and Antimicrobial Resistance. *mBio* **2018**, *9*, doi:10.1128/mBio.02112-18.

6. Klemm, E.J.; Shakoor, S.; Page, A.J.; Qamar, F.N.; Judge, K.; Saeed, D.K.; Wong, V.K.; Dallman, T.J.; Nair, S.; Baker, S. Emergence of an Extensively Drug-Resistant *Salmonella enterica* Serovar Typhi Clone Harboring a Promiscuous Plasmid Encoding Resistance to Fluoroquinolones and Third-Generation Cephalosporins. *mBio* **2018**, *9*, e00105-00118.

28. Britto, C.D.; Dyson, Z.A.; Duchene, S.; Carter, M.J.; Gurung, M.; Kelly, D.F.; Murdoch, D.R.; Ansari, I.; Thorson, S.; Shrestha, S., et al. Laboratory and molecular surveillance of paediatric typhoidal Salmonella in Nepal: Antimicrobial resistance and implications for vaccine policy. *PLOS Neglected Tropical Diseases* **2018**, *12*, e0006408, doi:10.1371/journal.pntd.0006408.

29. Wong, V.K.; Baker, S.; Pickard, D.J.; Parkhill, J.; Page, A.J.; Feasey, N.A.; Kingsley, R.A.; Thomson, N.R.; Keane, J.A.; Weill, F.-X. Phylogeographical analysis of the dominant multidrug-resistant H58 clade of *Salmonella* Typhi identifies inter-and intracontinental transmission events. *Nature genetics* **2015**, *47*, 632-639.

30. Wong, V.K.; Baker, S.; Connor, T.R.; Pickard, D.; Page, A.J.; Dave, J.; Murphy, N.; Holliman, R.; Sefton, A.; Millar, M. An extended genotyping framework for *Salmonella enterica* serovar Typhi, the cause of human typhoid. *Nature communications* **2016**, *7*.

35. Seemann, T. Prokka: rapid prokaryotic genome annotation. *Bioinformatics* **2014**, *30*, 2068-2069, doi:10.1093/bioinformatics/btu153.

51. Rahman, S.I.A.; Dyson, Z.A.; Klemm, E.J.; Khanam, F.; Holt, K.E.; Chowdhury, E.K.; Dougan, G.; Qadri, F. Population structure and antimicrobial resistance patterns of Salmonella Typhi isolates in urban Dhaka, Bangladesh from 2004 to 2016. *PLOS Neglected Tropical Diseases* **2020**, *14*, e0008036, doi:10.1371/journal.pntd.0008036.

52. Li, H.; Durbin, R. Fast and accurate short read alignment with Burrows–Wheeler transform. *bioinformatics* **2009**, *25*, 1754-1760.

53. Li, H.; Handsaker, B.; Wysoker, A.; Fennell, T.; Ruan, J.; Homer, N.; Marth, G.; Abecasis, G.; Durbin, R. The sequence alignment/map format and SAMtools. *Bioinformatics* **2009**, *25*, 2078-2079.

54. Li, H. A statistical framework for SNP calling, mutation discovery, association mapping and population genetical parameter estimation from sequencing data. *Bioinformatics* **2011**, *27*, 2987-2993.

55. Danecek, P.; Schiffels, S.; Durbin, R. Multiallelic calling model in bcftools (-m). June: 2014.

56. Parkhill, J.; Dougan, G.; James, K.; Thomson, N.; Pickard, D.; Wain, J.; Churcher, C.; Mungall, K.; Bentley, S.; Holden, M. Complete genome sequence of a multiple drug resistant *Salmonella enterica* serovar Typhi CT18. *Nature* **2001**, *413*, 848-852.

57. Lange, S.J.; Alkhnbashi, O.S.; Rose, D.; Will, S.; Backofen, R. CRISPRmap: an automated classification of repeat conservation in prokaryotic adaptive immune systems. *Nucleic Acids Res* **2013**, *41*, 8034-8044, doi:10.1093/nar/gkt606.

58. Zankari, E.; Hasman, H.; Cosentino, S.; Vestergaard, M.; Rasmussen, S.; Lund, O.; Aarestrup, F.M.; Larsen, M.V. Identification of acquired antimicrobial resistance genes. *J Antimicrob Chemother* **2012**, *67*, 2640-2644, doi:10.1093/jac/dks261.

59. El-Gebali, S.; Mistry, J.; Bateman, A.; Eddy, S.R.; Luciani, A.; Potter, S.C.; Qureshi, M.; Richardson, L.J.; Salazar, G.A.; Smart, A. The Pfam protein families database in 2019. *Nucleic acids research* **2018**, *47*, D427-D432.

60. Crooks, G.E.; Hon, G.; Chandonia, J.-M.; Brenner, S.E. WebLogo: a sequence logo generator. *Genome research* **2004**, *14*, 1188-1190.

61. Edgar, R.C. MUSCLE: multiple sequence alignment with high accuracy and high throughput. *Nucleic Acids Research* **2004**, *32*, 1792-1797, doi:10.1093/nar/gkh340.

62. Nguyen, L.-T.; Schmidt, H.A.; von Haeseler, A.; Minh, B.Q. IQ-TREE: A Fast and Effective Stochastic Algorithm for Estimating Maximum-Likelihood Phylogenies. *Molecular Biology and Evolution* **2015**, *32*, 268-274, doi:10.1093/molbev/msu300.

63. Kozlov, A.M.; Darriba, D.; Flouri, T.; Morel, B.; Stamatakis, A. RAxML-NG: a fast, scalable and user-friendly tool for maximum likelihood phylogenetic inference. *Bioinformatics* **2019**, 10.1093/bioinformatics/btz305, doi:10.1093/bioinformatics/btz305.

64. Letunic, I.; Bork, P. Interactive Tree Of Life (iTOL) v4: recent updates and new developments. *Nucleic Acids Research* **2019**, *47*, W256-W259, doi:10.1093/nar/gkz239.

65. Astrin, J.J.; Höfer, H.; Spelda, J.; Holstein, J.; Bayer, S.; Hendrich, L.; Huber, B.A.; Kielhorn, K.-H.; Krammer, H.-J.; Lemke, M., et al. Towards a DNA Barcode Reference Database for Spiders and Harvestmen of Germany. *PLOS ONE* **2016**, *11*, e0162624, doi:10.1371/journal.pone.0162624.
