# Supplementary material for: CRISPR-Cas Diversity in Clinical Salmonella enterica Serovar Typhi Isolates from South Asian Countries
Source: Genes (Basel). 2020 Nov 18;11(11):1365. doi: 10.3390/genes11111365 (PMC7698835; doi:10.3390/genes11111365)
Supplement: Supplementary file 1 [file genes-11-01365-s001.zip › Table S1 - S4 Final.docx]

**Table S1.** Different *S.* Typhi genotypes identified in this study.

| **Genotype** | **Frequency** | **Percentage (%)** |
| --- | --- | --- |
| 1.2.1 | 2 | 0.19 |
| 2 | 23 | 2.17 |
| 2.0.1 | 7 | 0.66 |
| 2.1.7 | 8 | 0.76 |
| 2.2 | 15 | 1.42 |
| 2.2.1 | 1 | 0.09 |
| 2.2.2 | 4 | 0.38 |
| 2.2.4 | 1 | 0.09 |
| 2.3.3 | 20 | 1.89 |
| 2.3.4 | 2 | 0.19 |
| 2.4 | 1 | 0.09 |
| 2.5 | 7 | 0.66 |
| 3 | 4 | 0.38 |
| 3.0.1 | 3 | 0.28 |
| 3.0.2 | 3 | 0.28 |
| 3.1 | 1 | 0.09 |
| 3.1.2 | 1 | 0.09 |
| 3.2.1 | 2 | 0.19 |
| 3.2.2 | 73 | 6.89 |
| 3.3 | 10 | 0.94 |
| 3.3.1 | 9 | 0.85 |
| 3.3.2 | 50 | 4.72 |
| 3.3.2.Bd1 | 21 | 1.98 |
| 3.3.2.Bd2 | 24 | 2.27 |
| 4.1 | 7 | 0.66 |
| 4.3.1 | 50 | 4.72 |
| 4.3.1.1 | 298 | 28.14 |
| 4.3.1.1.P1 | 88 | 8.31 |
| 4.3.1.2 | 213 | 20.11 |
| 4.3.1.3 | 55 | 5.19 |
| 4.3.1.3q1 | 56 | 5.29 |
| **Total** | **1059** | **100** |

**Table S2.** Estimated distance within and between different *Salmonella* serovars including *S.* Typhi based on multiple sequence alignment of all group-A CRISPR loci sequences.

| **Taxon** | **Mean_loci_length** | **#_of_intrasp_distances** | **Intrasp_dist_Mean** | **Intrasp_dist_Min** | **Intrasp_dist_Median** | **Intrasp_dist_Max** | **Closest_Sp** | **Closest_Sp_Min_Dist** | **Closest_Sp_Max_Dist** | **Closest_Sp_Dist_Median** | **#_distances_to_Closest_Sp** |
| --- | --- | --- | --- | --- | --- | --- | --- | --- | --- | --- | --- |
| *E*. *coli* | 783 | 42 | 6.54 | 2.04 | 6.66 | 13.4 | *S. enterica* Enterica Poona | 1.15 | 22.05 | 4.92 | 28 |
| *S. enterica* Typhi | 411 | 1109862 | 0.07 | 0 | 0 | 5.58 | *S. enterica* | 0.62 | 34.75 | 3.12 | 16864 |
| *S. enterica* Diarizonae | 2042 | 2 | 19.04 | 19.04 | 19.04 | 19.04 | *S. enterica* Enterica Newport | 2.56 | 17.12 | 13.73 | 10 |
| *S. enterica* Enterica Cubana | 1342 | 2 | 6.36 | 6.36 | 6.36 | 6.36 | *S. enterica* Enterica Dublin | 1.22 | 4.03 | 2.45 | 4 |
| *S. enterica* Enterica Dublin | 302 | 2 | 0.29 | 0.29 | 0.29 | 0.29 | *S. enterica* Enterica Enteritidis | 0.78 | 2.35 | 1.3 | 12 |
| *S. enterica* Enterica Enteritidis | 609 | 30 | 2.84 | 0 | 3.68 | 4.7 | *S. enterica* | 0 | 23.67 | 5.55 | 96 |
| *S. enterica* Enterica Gallinarum | 640 | 2 | 0.1 | 0.1 | 0.1 | 0.1 | *S. enterica* Enterica Dublin | 1.35 | 1.81 | 1.58 | 4 |
| *S. enterica* Enterica Heidelberg | 885 | 30 | 6.14 | 0 | 6.11 | 10.65 | *S. enterica* Enterica Typhimurium | 1.4 | 13.37 | 7.07 | 48 |
| *S. enterica* Enterica India | 578 | 0 |  |  |  |  | *S. enterica* Enterica Panama | 0 | 0 | 0 | 1 |
| *S. enterica* Enterica Indiana | 1066 | 2 | 9.44 | 9.44 | 9.44 | 9.44 | *S. enterica* | 0.02 | 29.85 | 10.35 | 32 |
| *S. enterica* Enterica Infantis | 1548 | 12 | 10.37 | 3.6 | 10.67 | 16.67 | *S. enterica* Enterica Dublin | 1.56 | 3.59 | 2.67 | 8 |
| *S. enterica* Enterica Johannesburg | 1005 | 0 |  |  |  |  | *S. enterica* Typhi | 1.8 | 8.24 | 2.91 | 1054 |
| *S. enterica* Enterica Kentucky | 1543 | 12 | 13.39 | 8.65 | 13.23 | 19.37 | *S. enterica* Enterica Newport | 1.7 | 18.19 | 11.05 | 20 |
| *S. enterica* Enterica Newport | 1090 | 20 | 7.44 | 1.88 | 9.06 | 12.9 | *S. enterica* Enterica Dublin | 0.83 | 3.2 | 2.25 | 10 |
| *S. enterica* Enterica Panama | 578 | 0 |  |  |  |  | *S. enterica* Enterica India | 0 | 0 | 0 | 1 |
| *S. enterica* Enterica ParaTyphi | 462 | 72 | 1.52 | 0 | 1.63 | 4.11 | *S. enterica* Enterica Dublin | 0.93 | 2.06 | 1.49 | 18 |
| *S. enterica* Enterica Poona | 1158 | 12 | 3.1 | 0 | 3.35 | 5.19 | *S. enterica* Enterica Newport | 0.96 | 18.34 | 2.74 | 20 |
| *S. enterica* Enterica Schwarzengrund | 913 | 2 | 6.81 | 6.81 | 6.81 | 6.81 | *S. enterica* Enterica Newport | 1.64 | 11.77 | 7.6 | 10 |
| *S. enterica* Enterica Typhimurium | 1143 | 56 | 8.49 | 0.45 | 8.05 | 15.08 | *S. enterica* Enterica | 1.25 | 12.55 | 5.58 | 56 |
| *S. enterica* Enterica | 717 | 42 | 4.08 | 1.43 | 2.76 | 8.44 | *S. enterica* | 0.94 | 29.07 | 5.68 | 112 |
| *S. enterica* | 1497 | 240 | 12.45 | 0 | 10.3 | 35.34 | *S. enterica* Enterica Enteritidis | 0 | 23.67 | 5.55 | 96 |

**Table S3.** Presence of multiple DR-spacer pairs in different genotypes, countries, and study settings.

| **Spacer-DR pair** | **Remarks** |
| --- | --- |
| Ts34d-Td35a | Specific to group-B loci, mostly present among the surveillance (n=154; P >0.05) and travel (n=36; P <0.001) related isolates. But it is nearly absent among Bangladeshi surveillance (n=6; P <0.001) and Pakistani outbreak (n=2, one XDR and one non-XDR, P <0.001) isolates (Fig. 2-3 and Table 2-3). |
| Ts55a-Td23a | Specific to group-B loci, prevalent among surveillance (n=283; P <0.001), travel (n=72; P <0.001) and outbreak (n=99; P <0.001) related isolates, but almost absent in Bangladesh (n=3; P <0.001) (Fig. 2-3 and Table 2-3). |
| Ts54a-Td39a/ Td39b | Specific to group-B loci, mostly present among the surveillance isolates (n=171; P <0.001), but nearly absent in outbreak (n=7; P <0.01) and travel (n=0; P <0.001) cases (Fig. 2-3 and Table 2-3). |

**Table S4.** Details on different copies of *DinG*, *DEDDh* and *WYL* genes. The length of the different copies of the genes was determined and added with their gene name (in superscript), to define an identifier for the CDS. An asterisk (*) was added to all *cas* genes of an isolate if the coding sequence (CDS) had any non-sense mutation and interrupted prematurely.

| **Gene** | **Copy name** | **Copy length (bp)** | **Present in Isolates** | **Match (Makarova et al 2018 database)** | **Match (*S.* Typhi CT18)** | **Other names of the protein** | **Remarks** |
| --- | --- | --- | --- | --- | --- | --- | --- |
| *DinG* | *DinG^*924^* | 924 | 1059 | COG1199_16764184 (697 aa) | STY0856 (pseudogene) |  | Degenerated and parts of the same pseudogene and always located on the same contig, separated by a nine base-pair sequence which contains a non-sense mutation. |
|  | *DinG^*1212^* | 1212 | 1059 | COG1199_16764184 (697 aa) | STY0856 (pseudogene) |  |  |
|  | *DinG^1911^* | 1911 | 1059 | COG1199_16765162 (632 aa) | STY1951 | ATP-dependent helicase | Located on a different contig than the first two and can produce a 636 aa long protein. |
| *DEDDh* | *DEDDh^546^* | 546 | 1059 | cd06127_158428766 |  | Oligoribonuclease |  |
|  | *DEDDh^648^* | 648 | 1059 | cd06127_90109308 |  | Ribonuclease T |  |
|  | *DEDDh^585^* | 585 | 1051 | cd06127_16763100 |  | Exodeoxyribonuclease |  |
|  | *DEDDh^558^* | 558 | 284 | cd06127_18466779 |  | DNA polymerase III PolC-type | Present in subtypes of genotype 3.3.2 and 4.3.1, but completely missing in 4.3.1.1.P1 and 4.3.1.3q1 |
| *WYL* | *WYL^693^* | 693 | 1058 | COG2378_16763790 | STY0448 | *DeoR* family transcriptional regulator |  |
|  | *WYL^681^* | 681 | 3 | COG2378_1723630 |  | hypothetical protein | Present in genotype 1.2.1 and 2.2.1 (three isolates) |
|  | *WYL^735^* | 735 | 1 | COG2378_16763790 |  | hypothetical protein |  |
|  | *WYL^888^* | 888 | 142 | COG2378_15794103 (88% coverage and 35% identity) | No match | hypothetical protein | Only present in genotype 4.3.1.3.Bdq and 4.3.1.1.P1 (56 and 86 isolates respectively). Match with plasmid pK91 (found in S. Typhi genotype 4.3.1.3q1), plasmid-2 of the XDR (genotype 4.3.1.1.P1) isolates from Pakistan and pCTXM-2248 of an *E. coli* (accession: MG836696.1) |
