# Supplementary figures and images for: CRISPR-Cas Diversity in Clinical Salmonella enterica Serovar Typhi Isolates from South Asian Countries
Source: Genes (Basel). 2020 Nov 18;11(11):1365. doi: 10.3390/genes11111365 (PMC7698835; doi:10.3390/genes11111365)

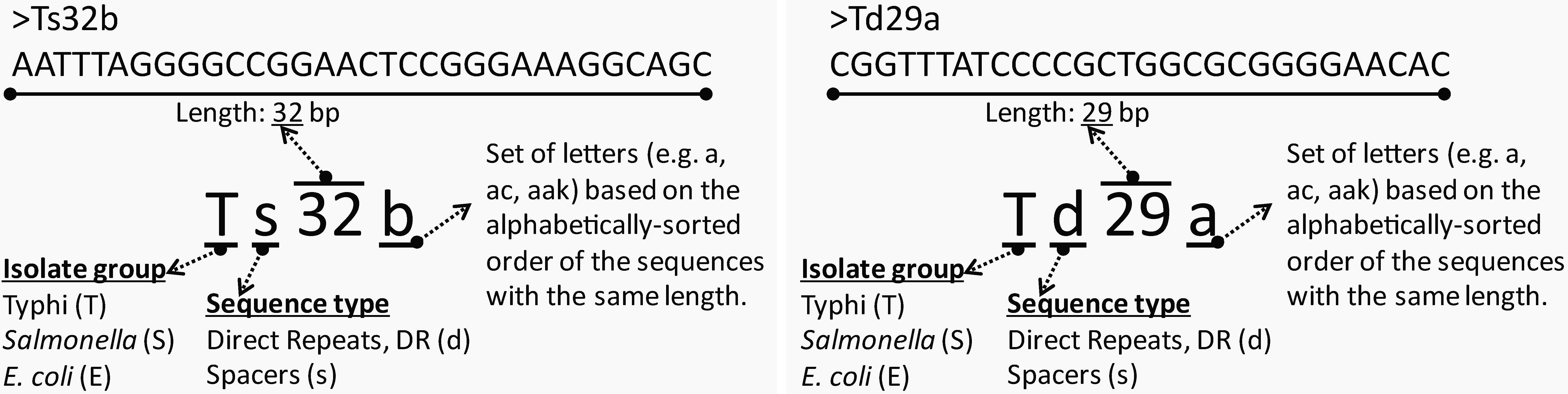

Supplement: Supplementary file 1 [file genes-11-01365-s001.zip › Figure S1_Identifier for DR and Spacers.tif]

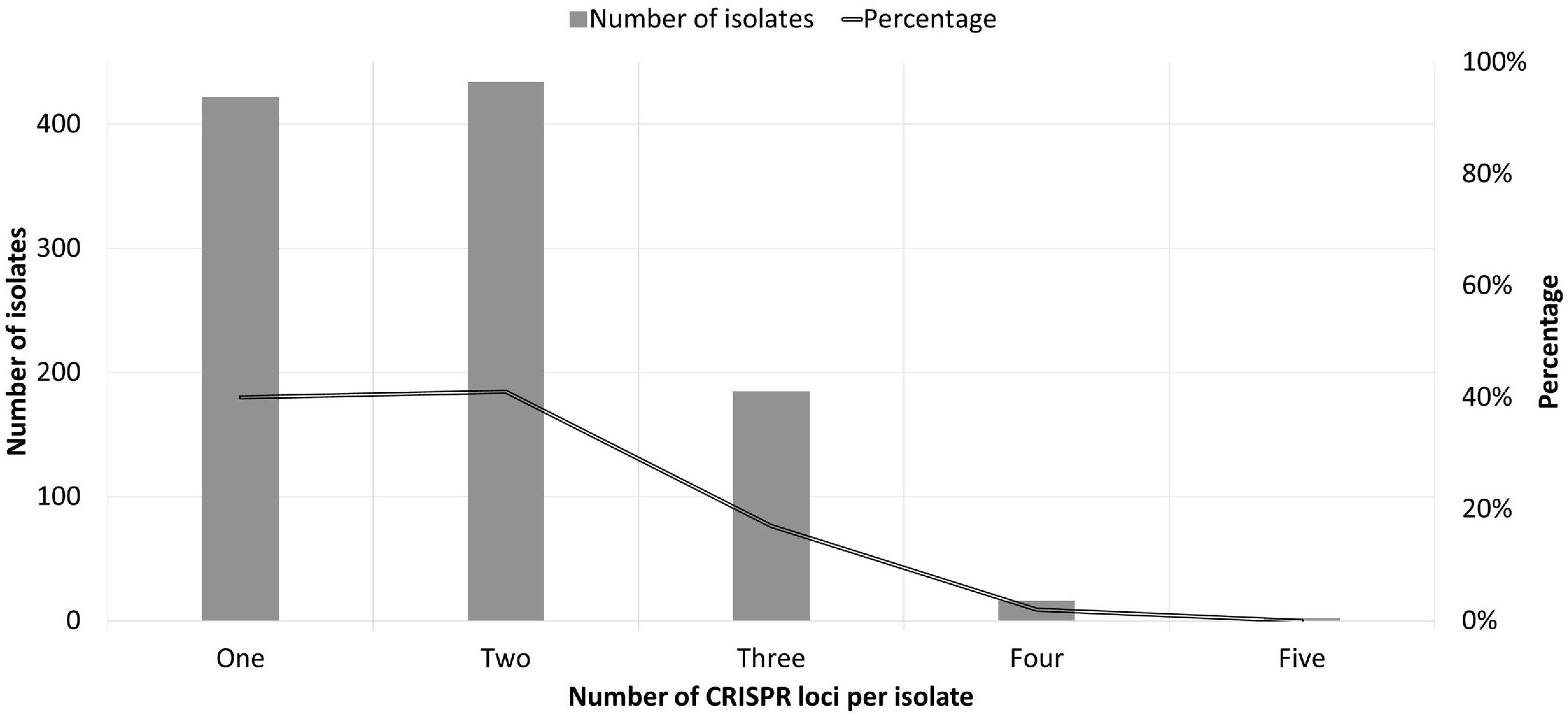

Supplement: Supplementary file 1 [file genes-11-01365-s001.zip › Figure S2_Loci_per_isolate.tif]

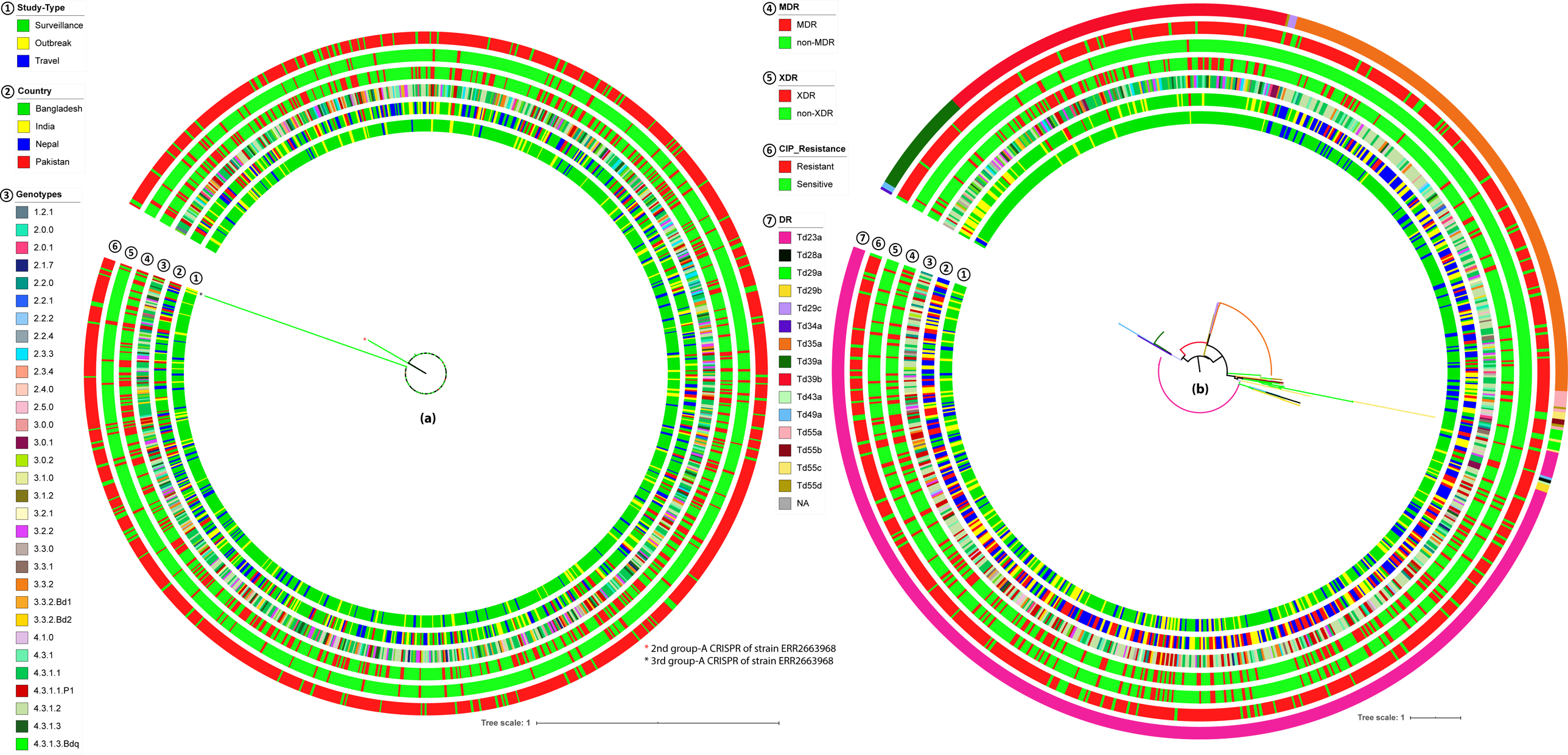

Supplement: Supplementary file 1 [file genes-11-01365-s001.zip › Figure S3_All_Typhi_CRISPRs v3.tif]

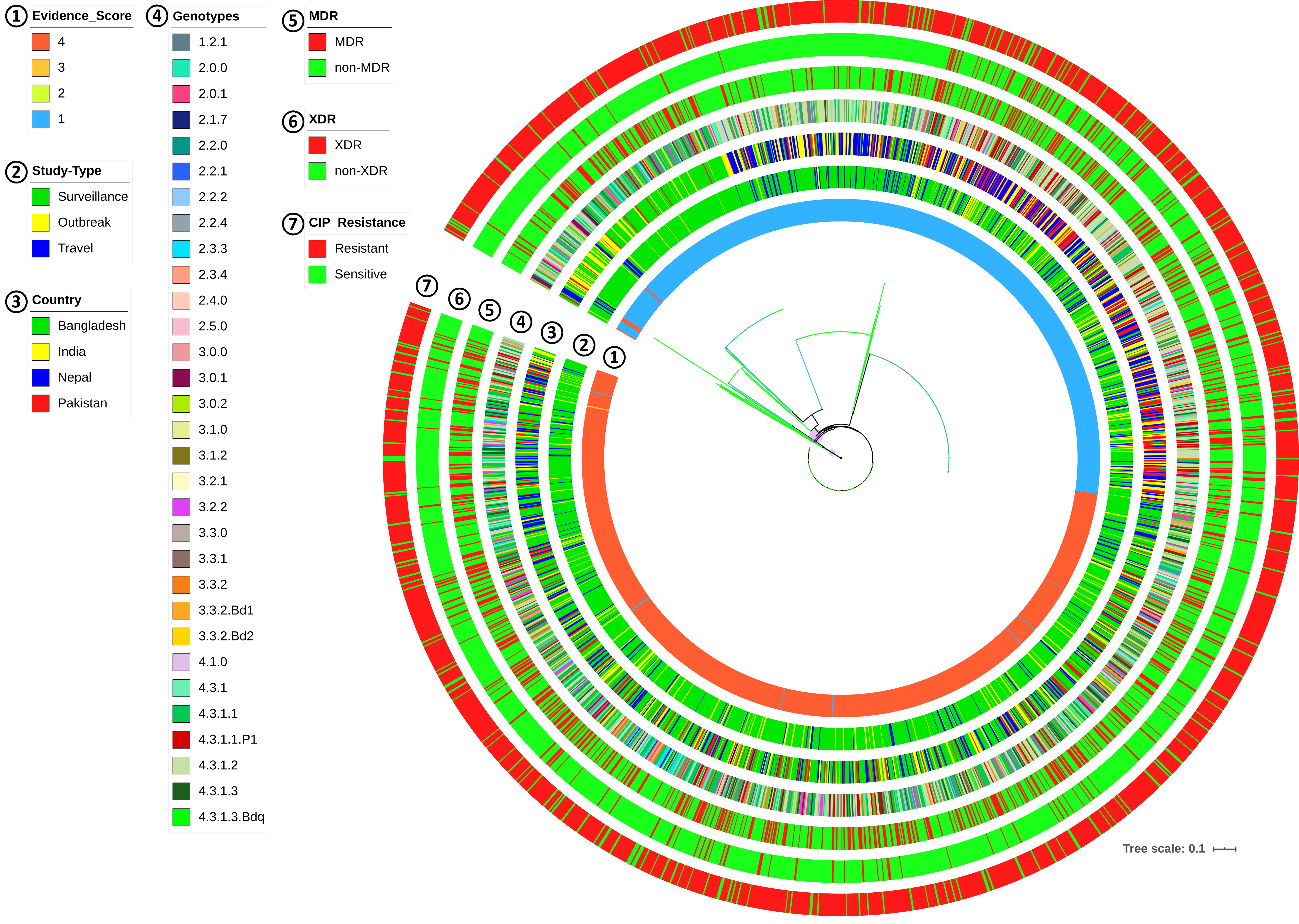

Supplement: Supplementary file 1 [file genes-11-01365-s001.zip › Figure S4_All_Typhi_group-A+B_CRISPRs_v2.tif]

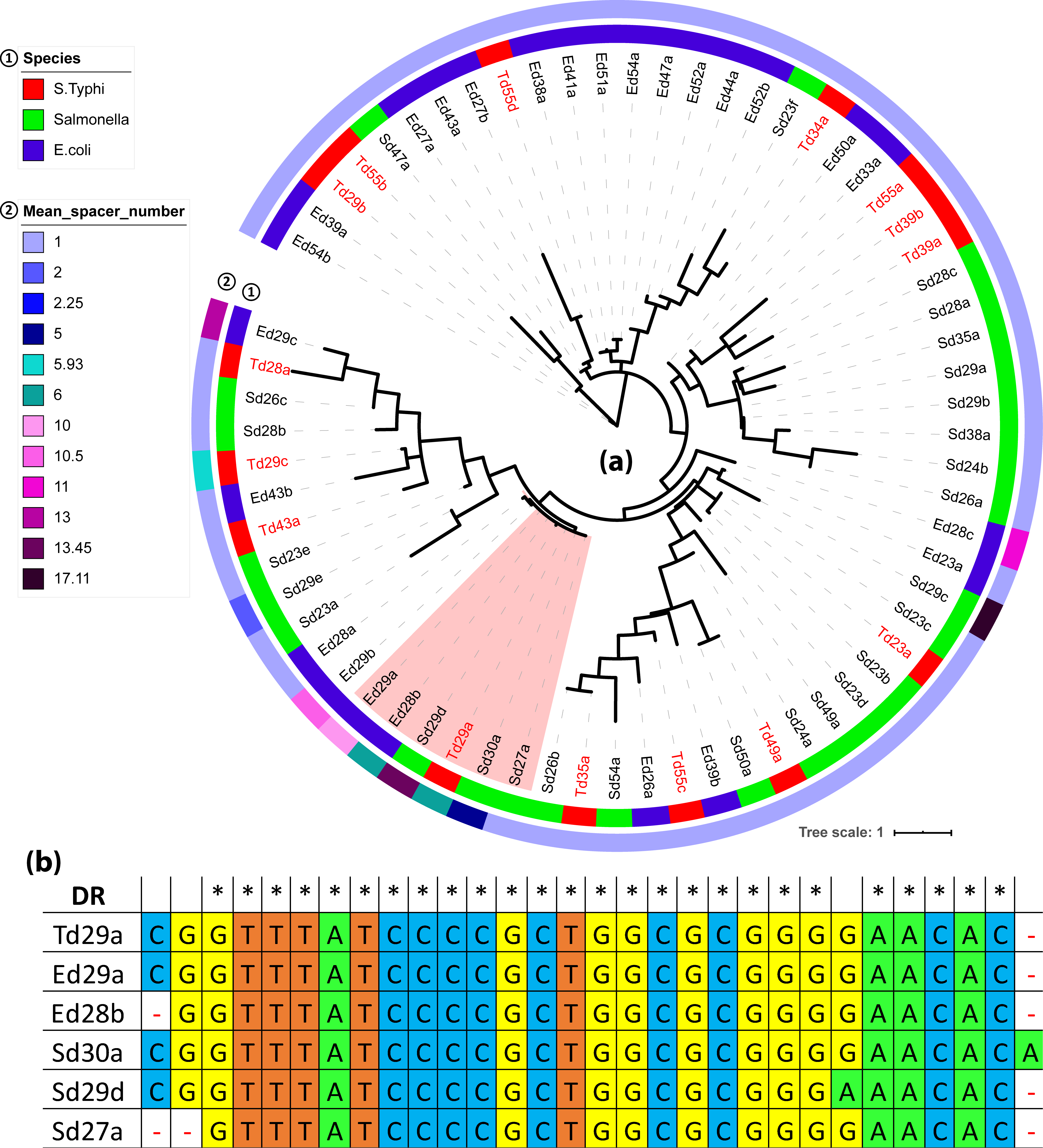

Supplement: Supplementary file 1 [file genes-11-01365-s001.zip › Figure S5_All_DR_seq_reviewed v1.tif]

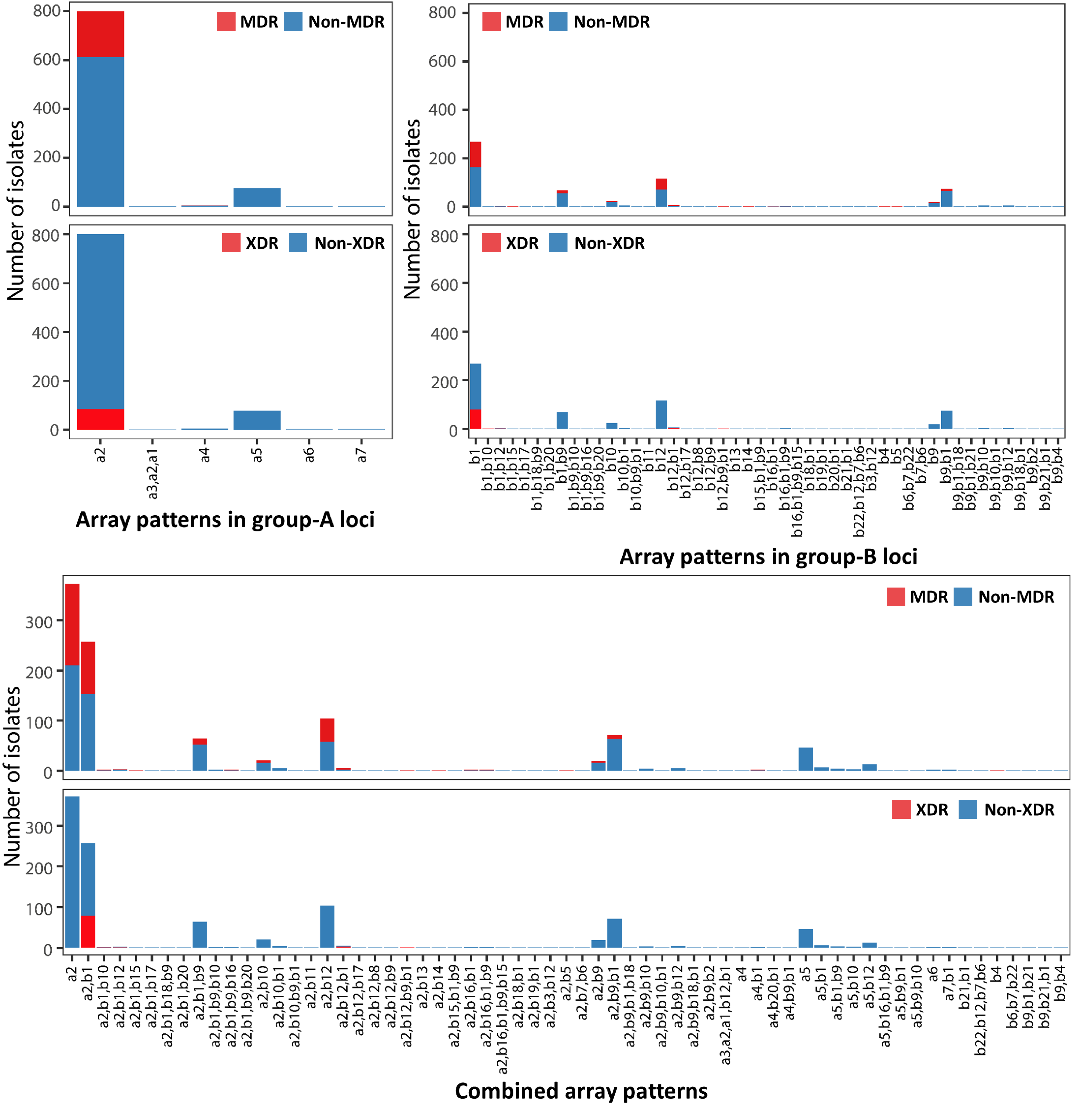

Supplement: Supplementary file 1 [file genes-11-01365-s001.zip › Figure S6.tif]

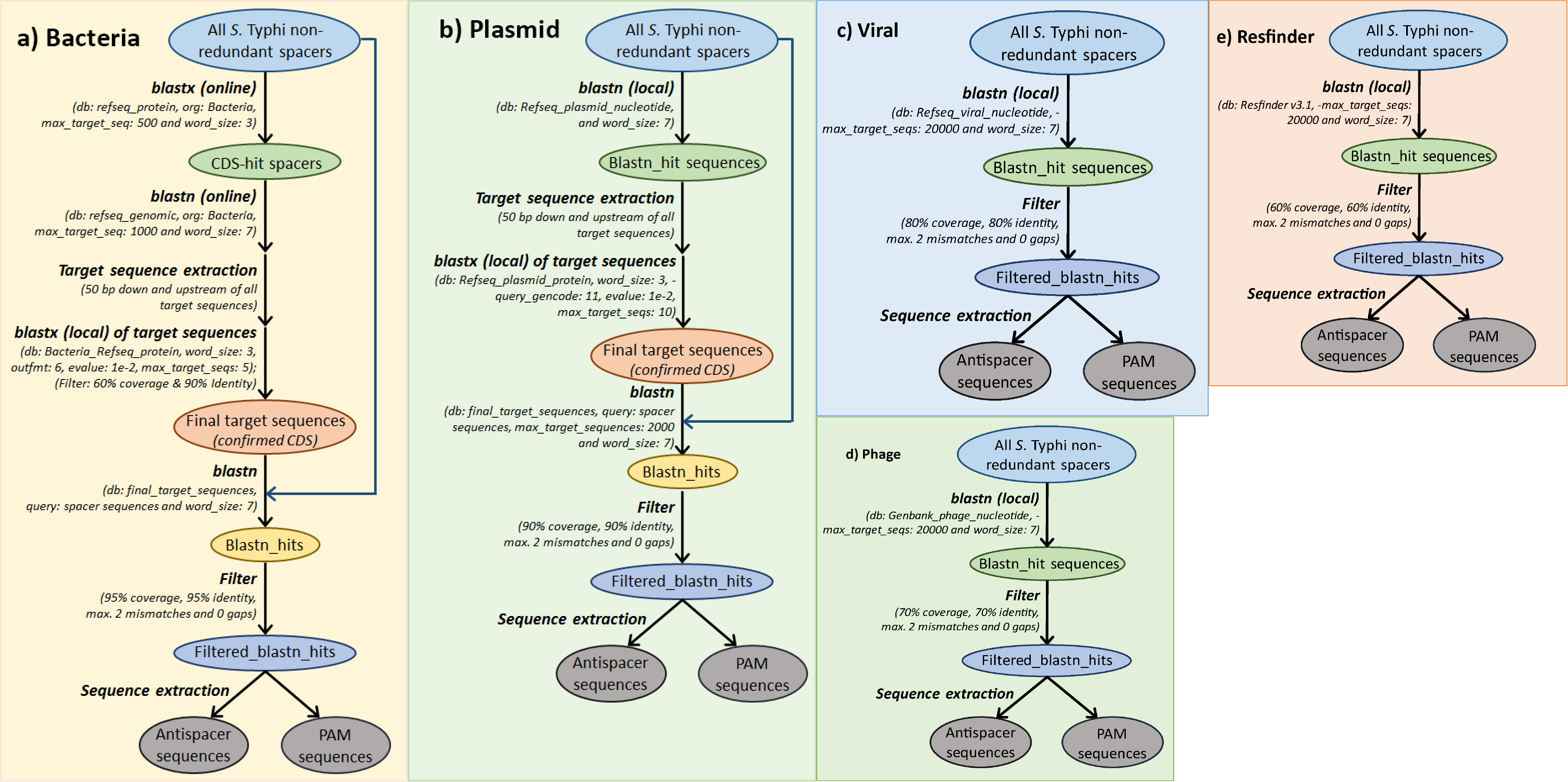

Supplement: Supplementary file 1 [file genes-11-01365-s001.zip › Figure S7_Spacer_Target_method.tif]

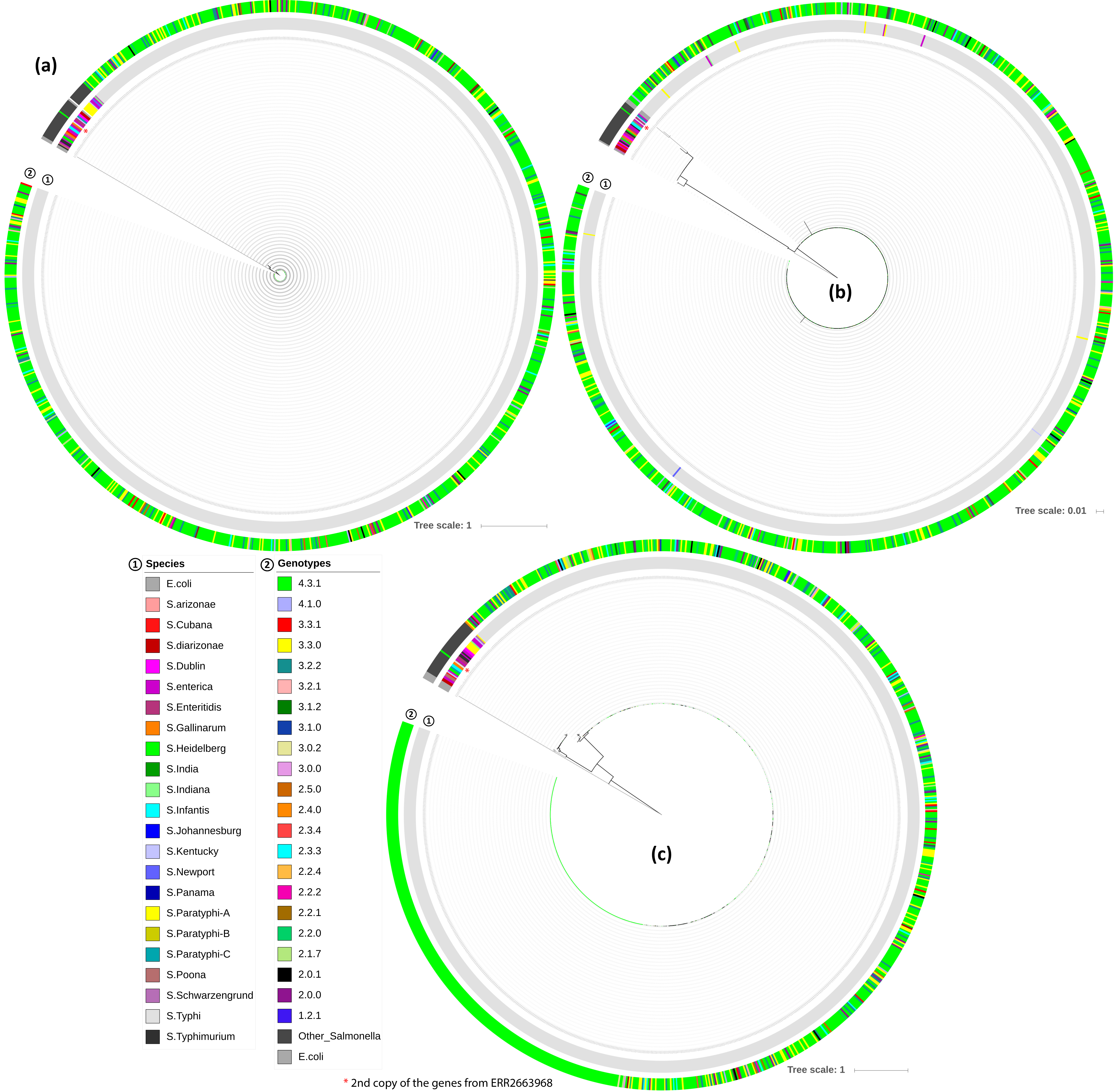

Supplement: Supplementary file 1 [file genes-11-01365-s001.zip › Figure S8_All_Cas.tif]

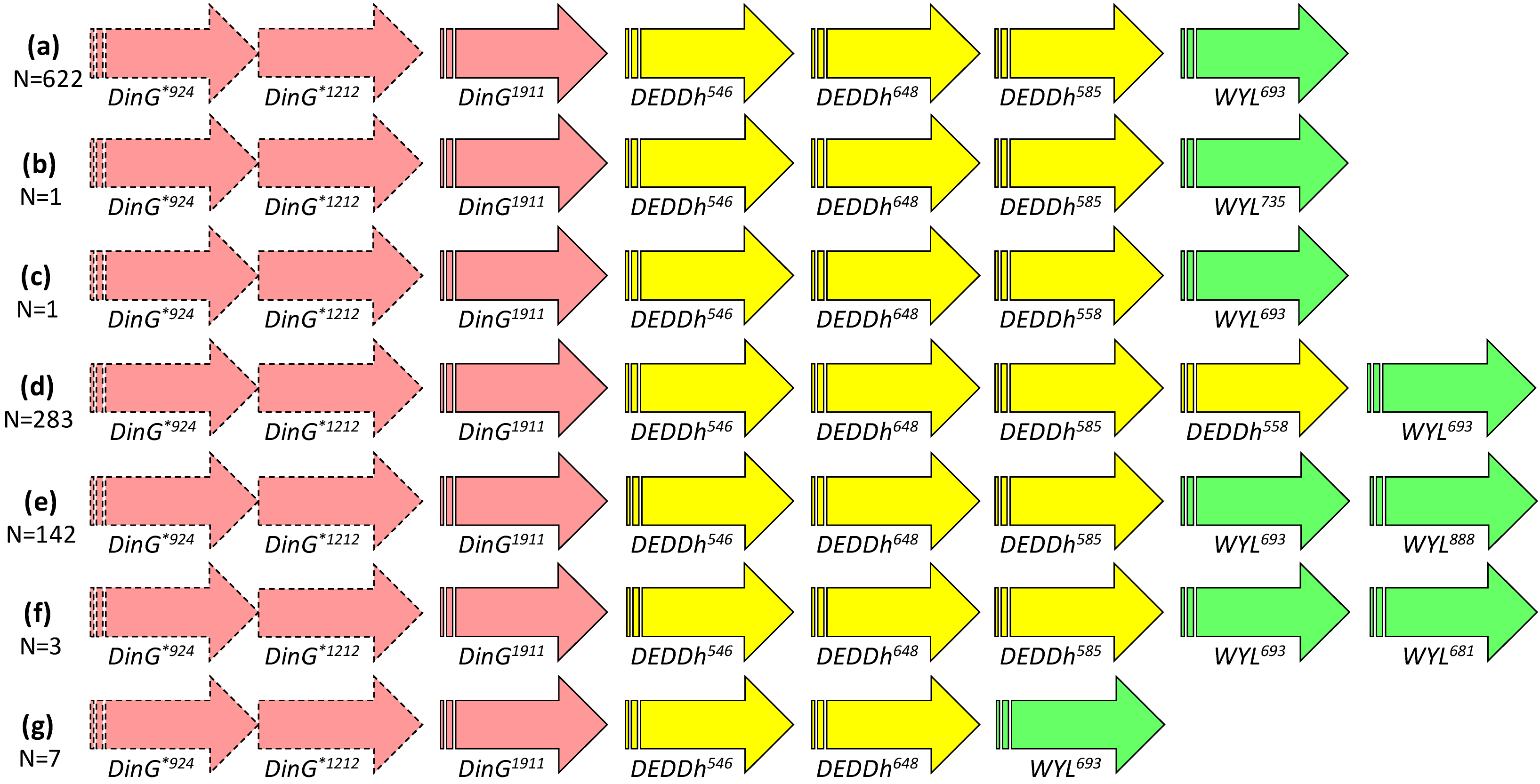

Supplement: Supplementary file 1 [file genes-11-01365-s001.zip › Figure S9_DinG_DEDDh_WYL v3.tif]
